# Loss of nsp14-exonuclease activity impairs the replication, proofreading, fitness, and pathogenesis of SARS-CoV-2

**DOI:** 10.1128/mbio.00073-26

**Published:** 2026-05-06

**Authors:** Jordan Anderson-Daniels, Meghan V. Diefenbacher, Boyd L. Yount, Rita M. Meganck, Longping V. Tse, Kaitlyn N. Burke, Hector A. Miranda, D. Trevor Scobey, Xiaotao Lu, Laura Stevens, Kenneth H. Dinnon, Nathaniel S. Chapman, Camryn Pajon, John M. Powers, Cameron Nguyen, Rachel L. Graham, Nicholas S. Heaton, Ralph S. Baric, Mark R. Denison, Timothy P. Sheahan

**Affiliations:** 1 Department of Pediatrics, Vanderbilt University Medical Center 12328, Nashville, Tennessee, USA; 2 Department of Epidemiology, University of North Carolina at Chapel Hill 154805, Chapel Hill, North Carolina, USA; 3 Department of Molecular Microbiology, Washington University in St Louis 169015, St. Louis, Missouri, USA; 4 Department of Molecular Microbiology and Immunology, Saint Louis University 573679, St. Louis, Missouri, USA; 5 Department of Molecular Genetics and Microbiology, Duke University School of Medicine 12277 , Durham, North Carolina, USA; 6 Department of Microbiology and Immunology, University of North Carolina at Chapel Hill 318275, Chapel Hill, North Carolina, USA; 7 Department of Pathology, Microbiology, and Immunology, Vanderbilt University Medical Center 204907, Nashville, Tennessee, USA; The University of Iowa, Iowa City, Iowa, USA

**Keywords:** viral pathogenesis, viral replication, coronavirus, innate immunity, RNA virus

## Abstract

Coronaviruses (CoVs) replicate their RNA genomes with a higher degree of fidelity than other RNA viruses, a mechanism mediated by the proofreading and recombination activities of the exoribonuclease domain of replicase nonstructural protein 14 (nsp14-ExoN). Both murine hepatitis virus (MHV) and SARS-CoV tolerate nsp14-ExoN loss-of-function mutations (ExoN−) (D90A and E92A), but have impaired replication fidelity and pathogenesis; yet identical substitutions in MERS-CoV and SARS-CoV-2 have been reported to be lethal. Here, we report a saturation mutagenesis approach facilitating the recovery and analysis of several constellations of SARS-CoV-2 nsp14 ExoN-inactivating, loss-of-function substitutions, including the canonical D90A and E92A. Biochemical assays with purified WT or ExoN-nsp10-14 fusion proteins confirmed that active site substitutions abolished ExoN activity (ExoN−). SARS-CoV-2 ExoN− viruses exhibited impaired replication, RNA synthesis, and recombination, as well as decreased replication fidelity and loss of fitness *in vitro*. ExoN− viruses were significantly attenuated for replication in human primary airway epithelial cells and were attenuated for replication and pathogenesis in WT mice, as well as the highly susceptible K18 transgenic mice. In the absence of interferon signaling *in vivo*, SARS-CoV and SARS-CoV-2 ExoN− viral replication could be partially restored. These results demonstrate that SARS-CoV-2 ExoN− viruses are viable but highly impaired for replication, fitness, and fidelity *in vitro,* as well as innate immune antagonism and pathogenesis *in vivo*. Collectively, our results solidify the multiple critical roles of nsp14-ExoN across CoV genera and establish new approaches for rescuing and analyzing loss-of-function substitutions in studies of CoV replication, pathogenesis, and evolution.

**IMPORTANCE:**

Coronaviruses (CoV) are important human pathogens causing hundreds of millions of infections and millions of deaths over the past 20 years. The study of how these viruses multiply and cause disease identifies points of attack for therapeutics. Using a high-throughput genetic approach, we systematically inactivated an essential enzyme CoV needs for replication called ExoN. We show that without ExoN, CoV replication fidelity and fitness are reduced in cell culture. Replication without ExoN in mice was diminished but could be partially restored in mice that lack key components of the immune response. Altogether, we reveal new insights into the complexities of CoV replication and virus and host interactions, which could be leveraged for the development of novel multifaceted therapeutics that attack the ever-expanding functions of the CoV replication complex in replication and pathogenesis

## INTRODUCTION

Coronaviruses (CoVs) are etiologic agents of both endemic and pandemic human disease. Three novel CoVs have emerged in the past 20 years, including severe acute respiratory syndrome CoV (SARS-CoV), Middle East respiratory syndrome CoV (MERS-CoV), and SARS-CoV-2, the causative agent of the recent COVID-19 pandemic. All human CoVs (HCoVs) are thought to have originated from zoonoses (1–5). Given the great diversity of CoVs in animal reservoir species, future spillover events may give rise to novel human diseases. Thus, understanding the mechanisms by which CoVs replicate and evolve is critical for the understanding of emergence potential and the treatment and prevention of current and future emerging CoV infections. Much of the success in the development of COVID-19 antiviral therapies has resulted from the many viral, biochemical, and structural studies of the CoV replication-transcription complex (RTC). The CoV RTC is assembled from enzymatic and non-enzymatic cofactor proteins, including the non-structural proteins 7–16 (nsp7–16), where the nsp12 RNA-dependent RNA polymerase (nsp12-RdRP), along with cofactors nsp7 and nsp8, drives genome replication, sub-genomic mRNA synthesis, and initial guanylyl-transferase activity to initiate RNA capping (5–7). CoVs and other members of the order *Nidovirales* encode a 3′ to 5′ exoribonuclease (nsp14-ExoN) that regulates replication fidelity through RNA-dependent RNA proofreading functions during RNA synthesis (8–11). RNA proofreading has been proposed as a critical determinant of nidovirus evolution, pathogenesis, and the maintenance of their large RNA genomes while using an otherwise low-fidelity RdRp (9, 12–14). ExoN is a shared motif in all nidoviruses with genomes greater than 20 kb, and recent transcriptome mining has identified nidoviruses with genomes as large as 64 kb, twice that of SARS-CoV-2 (9, 15–18). Thus, nsp14-ExoN is a central determinant of CoV replication fidelity and evolutionary potential.

The first evidence that nidoviruses encoded an ExoN domain was identified through bioinformatic approaches with SARS-CoV nsp14. SARS-CoV nsp14 ExoN activity was subsequently confirmed in biochemical experiments (10, 19). Nsp14-ExoN is a member of the dnaQ-like DE-D-DH family of RNA and DNA exonucleases (20). The CoV ExoN contains three metal-coordinating active site motifs (i.e., motif I-Asp-X-Glu [DE], motif II-Glu [E], and motif III-Asp-His [DH]), which give the DE-D-DH family its name, although CoVs have a unique substitution in motif II, making their active site residue set DE-E-DH (10). ExoN hydrolyzes ssRNA and dsRNA in a 3′−5′ direction and excises 3′ single-nucleotide mismatches in template RNAs, activities that require binding of the nonenzymatic nsp10 cofactor (10, 21–23). Alanine substitutions of MHV and SARS-CoV motif I (DE to AA) (nsp14-ExoN−) result in significant defects in RNA replication fidelity and proofreading, thus are “loss of function” (LOF) mutations (12, 13). Importantly, ExoN-inactivating mutations attenuate replication and pathogenesis in mice, but the underlying mechanisms are not completely understood (24).

While ExoN active site substitutions were tolerated in MHV and SARS-CoV, similar ExoN substitutions in other CoVs, including HCoV-229E, MERS-CoV, and SARS-CoV-2, have not been viable despite these viruses sharing completely conserved active site domain motifs and highly conserved structures with SARS-CoV (10, 25, 26). Here, we report the recovery and analysis of SARS-CoV-2 viruses with nsp14-ExoN LOF substitutions (ExoN−) in the motif-I active site. Using a saturation mutagenesis approach, a highly permissive engineered Vero E6 cell line, and low temperature, we recovered a series of viable motif-I substitutions in SARS-CoV-2 ExoN. These experimental conditions also facilitated the recovery of a SARS-CoV-2 with classical motif-1 D90A/E92A substitutions. Biochemical assays confirmed that mutations found in viable viruses abolished ExoN enzymatic activity. SARS-CoV-2 ExoN− exhibited impaired replication, altered RNA synthesis, loss of competitive fitness, increased sensitivity to nucleoside analogs, decreased fidelity, and altered recombination patterns in the highly permissive and notably interferon (IFN) incompetent Vero cells. SARS-CoV-2 ExoN− viruses were highly attenuated for replication in primary human airway epithelial (HAE) cells and in WT and highly permissive K18 ACE2 transgenic mice. The attenuation of SARS-CoV-2 ExoN− replication could be partially reversed in mice lacking type I and type III IFN signaling. Altogether, these data indicate that *in vivo* attenuation of ExoN− viruses is not solely driven by general defects in replication but rather highlights the role of ExoN in innate immune antagonism and pathogenesis. Thus, our results with SARS-CoV-2 further establish that LOF nsp14-ExoN− mutations result in highly conserved biochemical, virological, and pathogenic defects across divergent CoVs. Finally, these studies demonstrate the essential role of nsp14-ExoN in CoV replication, pathogenesis, and immune evasion, making it an ideal target for broadly active therapeutic interventions.

## RESULTS

### SARS-CoV-2 nsp14-ExoN mutants are viable but attenuated for replication *in vitro*

**Fig 1:**
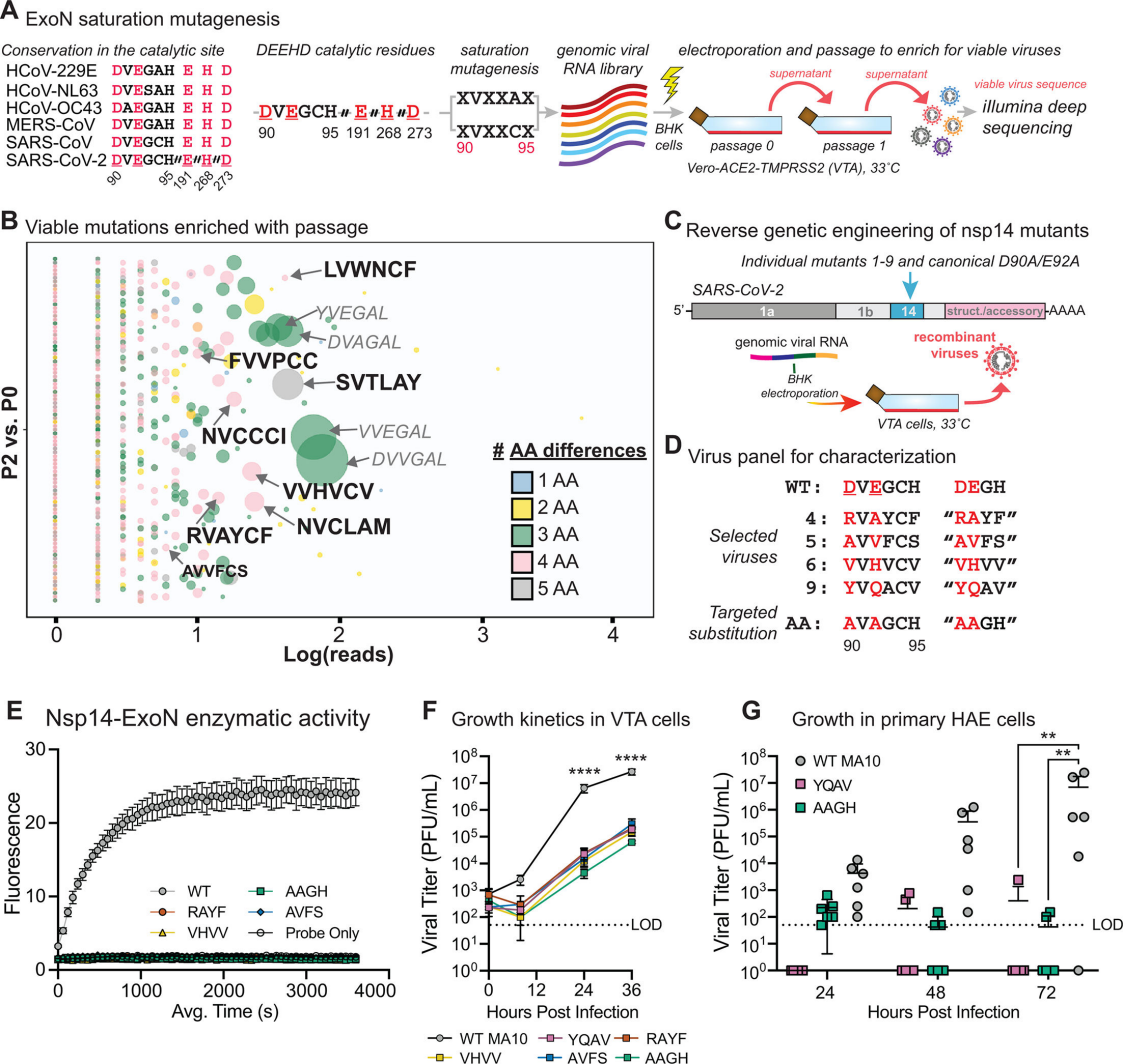
SARS-CoV-2 nsp14-ExoN mutants are viable but attenuated for replication *in vitro*. (**A**) Schematic of the saturation mutagenesis of ExoN motif-I. A viral RNA library with all possible combinations of amino acid mutations at positions 90, 92, 93, and 95 was electroporated into BHK cells and cocultured with VTA cells at 33°C. Viable viruses were enriched via passage on VTA cells and identified through Illumina deep sequencing. (**B**) Frequency of mutation series in viable genomes after passage. (**C**) Schematic for reverse genetic approach to engineer sets with four or more changes from WT, as well as the canonical D90A/E92A mutation into SARS-CoV-2 MA10. (**D**) List of substitutions present within the ExoN motif of panel viruses. (**E**) SARS-CoV-2 ExoN inactivating mutations abolish enzymatic activity. The *in vitro* exonuclease activity for select ExoN mutants was determined by a fluorescence RNA cleavage assay using nsp10/nsp14 fusion proteins. The RNA cleavage activity of WT and mutant nsp14 fusion proteins was measured over time using normalized amounts of protein and double-stranded RNA FRET probe. Symbols represent mean values and ± SEM error bars from four independent experiments. (**F**) Growth kinetics of SARS-CoV-2 MA10 ExoN mutant panel. VTA cells were infected at an MOI of 0.01, input removed, monolayers were rinsed once, and infectious virus in culture media was measured via plaque assay over time. Asterisks indicate significant differences (*P* < 0.0001) among mutant viruses and WT via two-way ANOVA Dunnett’s multiple comparison test. (**G**) Growth of select SARS-CoV-2 MA10 ExoN viruses and WT in primary HAE cell cultures. Data are combined from two independent studies with cells derived from two different human donors. HAE cells were infected at an MOI of 0.1 for 1.5 h after which input was removed, and cultures were washed with phosphate-buffered saline (PBS). Infectious virus production was measured via plaque assay of apical washes collected at the indicated times. Asterisks indicate statistical differences in titer via the Mann-Whitney test (YQAV *P* = 0.0054, AAGH *P* = 0.0076).

The SARS-CoV-2 nsp14-ExoN has five active site residues (D90, E92, E191, H268, and D273) in three distinct motifs (26). These active site residues are highly conserved among HCoVs (Fig. 1A). We previously demonstrated that alanine substitutions of motif-I catalytic residues (D90A, E92A) were viable in MHV and SARS-CoV but had diminished proofreading and fitness, yet other groups failed to recover similar mutants with MERS-CoV and SARS-CoV-2 (12, 13, 26). To maximize the potential for viable virus recovery and to comprehensively query the genetic plasticity of the SARS-CoV-2 nsp14-ExoN active site, we performed saturation mutagenesis of motif I active site residues, generating all possible single through quadruple amino acid coding combinations at residues D90, E92, G93, and H95 (WT-DEGH) (Fig. 1A). Since residue 94 is cysteine in SARS-CoV and SARS-CoV-2, but alanine in other HCoV, the saturation mutagenesis libraries were generated holding this residue constant as cysteine or alanine totaling 320,000 potential combinations (Fig. 1A). Pooled library genomes were electroporated into BHK cells and co-cultured with Vero E6 cells overexpressing SARS-CoV-2 entry factors human transmembrane serine protease 2 (TMPRSS2) and angiotensin-converting enzyme 2 (ACE2), hereafter referred to as “VTA” cells. Cells were incubated at 33°C, and clarified viral supernatants were passaged twice on naive VTA cells to enrich for viable viruses. After passage, viral RNA from supernatants was sequenced to determine the constellation of possible mutational patterns in infectious viral genomes (Fig. 1B). A total of 124 unique variants were detected (Table S1). Over half of the variants had D90 or E92 mutated, although no single mutants in either position were detected. In all, 47 variants were detected with both D90 and E92 mutated, all of which were also mutated at H95. A few of the variants that had both D90 and E92 mutated had changes that preserved the area’s negative charge, with one variant switching the D and E residues, and several others with a D or E present at other Motif I residues. In addition, several mutants contained positively charged (R, K, and H) or aromatic (Y and F) residues. All viable variants retained WT amino acid position V91. Position 95H was the most tolerant of change, with a wide variety of amino acid substitutions detected. WT (DVEGCH) variants were not detected, although sequences very close to WT (e.g., DVEGCT, DVEGCG) were detected at a low level (Table S1). In addition to the classical D90A/E92A substitutions in motif-I (AAGH), a subset of mutants that were enriched after passage and/or that had four or more changes in motif-I (90–95aa) were selected for rescue via directed mutagenesis in the SARS-CoV-2 MA10 reverse genetic system (Fig. 1C) (27). Our goal was to generate an initial panel of viruses that could be evaluated *in vitro* and *in vivo*; thus, this initial work was performed in the mouse-adapted SARS-CoV-2 MA10 background. Using our optimized virus recovery conditions (i.e., VTA cells, 33°C), robust recovery was achieved for four of the nine intended mutant viruses: RAYF, AVFS, VHVV, and YQAV (WT = DEGH) in addition to a virus containing the classical AAGH mutations (Fig. 1D). To gain insight into the impact of these motif-I mutations on nsp14-ExoN enzymatic activity and replication, we performed a series of biochemical and virologic studies. First, we assessed the exonuclease activity of select ExoN mutants in a FRET-based biochemical assay with nsp10-14 fusion protein, since nsp10 is a required cofactor of ExoN (28, 29). For this assay, the nsp-10-14 enzyme attacks a double-stranded RNA substrate with mismatched base pairing, liberating the fluorophore and quencher associated with the probe, resulting in measurable fluorescence. While WT nsp14/nsp10 exhibited notable exonuclease activity, none of the mutant enzymes assessed, including the canonical AAGH, had any detectable exonuclease activity (Fig. 1E). Thus, substitutions observed in viable SARS-CoV-2 MA10 viruses rendered the nsp14-ExoN enzyme catalytically dead. Second, we compared viral growth kinetics of the mutant panel and WT SARS-CoV-2 MA10 in VTA cells. All ExoN mutant panel viruses had significantly reduced replication compared to the WT virus, with ~2 log reduction in peak titers at 36 h post-infection (hpi) (Fig. 1F). To determine whether ExoN− viruses were attenuated in a more biologically relevant and IFN-competent culture system, we infected human primary airway epithelial (HAE) cell cultures with select mutant and WT viruses and monitored virus growth over time (Fig. 1G). HAE cells model the cellular complexity and architecture of the human conducting airway and contain epithelial subsets targeted by SARS-CoV-2 in humans (30, 31). Unlike the WT SARS-CoV-2 MA10 virus, which had increasing virus production over time, AAGH and YQAV viruses were significantly attenuated in HAE. Altogether, our data demonstrate that multiple constellations of ExoN motif I mutations are viable in SARS-CoV-2, but resultant viruses are attenuated for replication in Vero cells and significantly attenuated in primary human airway epithelial cells.

### SARS-CoV-2 ExoN mutations decrease specific infectivity

**Fig 2:**
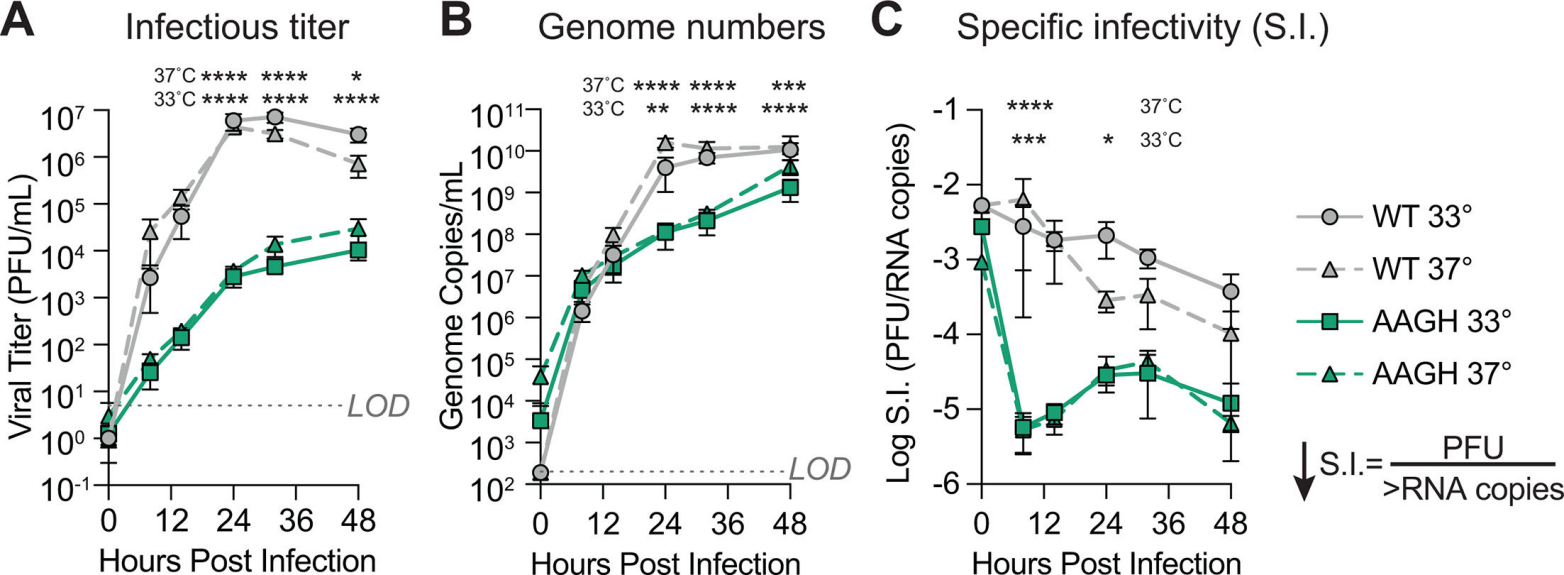
SARS-CoV-2 ExoN inactivating mutations abolish enzymatic activity and decrease specific infectivity. (**A**) Growth of SARS-CoV-2 WT or AAGH virus at 33°C or 37°C in VTA cells infected at an MOI of 0.01 PFU/cell. Titers were determined in supernatant by plaque assay at the indicated times. (**B**) Viral genome copy number in samples evaluated in panel **A** was determined by RT-qPCR. (**C**) Specific infectivity was determined as the ratio of genome copies/PFU from data shown in panels **A** and **B**. Graphed are mean values ± standard error of the mean (SEM) from triplicate infections of two independent experiments. Asterisks indicate statistical significance by two-way ANOVA with Sidak’s multiple comparison test.

Next, we sought to determine the impact of ExoN inactivation on SARS-CoV-2 replication, RNA synthesis, and specific infectivity. For these studies, we generated the canonical motif-I AAGH mutation in the SARS-CoV-2 WA/1 background, given the abundance of data we have previously generated with these mutations with MHV and SARS-CoV (12, 13, 32). First, we recovered WT and AAGH viruses at 33°C in VTA cells (P01) and then generated P1 stocks by passaging on VTA cells at either 33°C or 37°C. We then evaluated viral growth kinetics of these stocks at the temperature in which the stocks were prepared, normalizing virus input by infectious titers determined on VTA cells (MOI = 0.01). While recovery of ExoN− viruses was only achieved at 33°C, the levels and kinetics of replication were similar at both 33°C and 37°C for WT and AAGH viruses (Fig. 2A). Like SARS-CoV-2 MA10 ExoN− viruses, SARS-CoV-2 WA/1 AAGH virus was similarly attenuated for replication in VTA cells with significant differences from WT 24-48 hpi. Mean infectious titer differences between WT and AAGH viruses were greatest at 24 hpi, exceeding 3 logs for both temperature conditions. We then performed RT-qPCR to quantify viral genomic RNA in growth kinetic supernatants to better understand the relationship between infectious virus and virus particle production. In contrast to infectious titers, viral RNA copy number was similar for WT and AAGH viruses at early times but diverged beginning at 24 hpi, which continued through 48 hpi (Fig. 2B). Like infectious titer data, temperature did not have an appreciable difference on the numbers of secreted viral RNA genomes for WT and AAGH viruses (Fig. 2B). Using the viral RNA copy number (Fig. 2B) and infectious titer (Fig. 2A) data, we then calculated the specific infectivity (i.e., PFU/genome RNA copy ratio), a value which provides insight into the relative infectiousness of a virus sample. While the specific infectivity (S.I.) varied over time for both WT and AAGH virus samples, generally AAGH had a 10- to 100-fold decreased S.I. consistent with AAGH generating more non-infectious RNA or particles relative to WT virus (Fig. 2C). To understand the potential impact of ExoN inactivation on RNA synthesis, we performed a high MOI infection (MOI = 1) with WT and AAGH virus and quantified the genomic RNA 7 h after infection. Significantly less genomic RNA was produced by AAGH virus compared to WT, suggesting that genetic inactivation of ExoN attenuated RNA synthesis (Fig. S1A). Lastly, we determined the specific infectivity of the viral stocks used for the studies herein. Remarkably, the copies/mL of viral RNA were similar for all virus stocks assessed, yet the infectious titers for the ExoN mutant stocks were diminished from WT (Fig. S1B). In agreement with the growth kinetic data, the specific infectivity of ExoN-deficient viruses is compromised, but the impact on stock S.I. varied by ExoN mutation (Fig. S1B). Overall, these studies show temperature does not have a significant impact on WT or ExoN− viral replication once stocks are established, and that mutations inactivating SARS-CoV-2 nsp14-ExoN attenuate replication, RNA synthesis, and infectious virus production while increasing numbers of non-infectious particles generated.

### SARS-CoV-2 AAGH virus has increased sensitivity to nucleoside analogs

**Fig 3:**
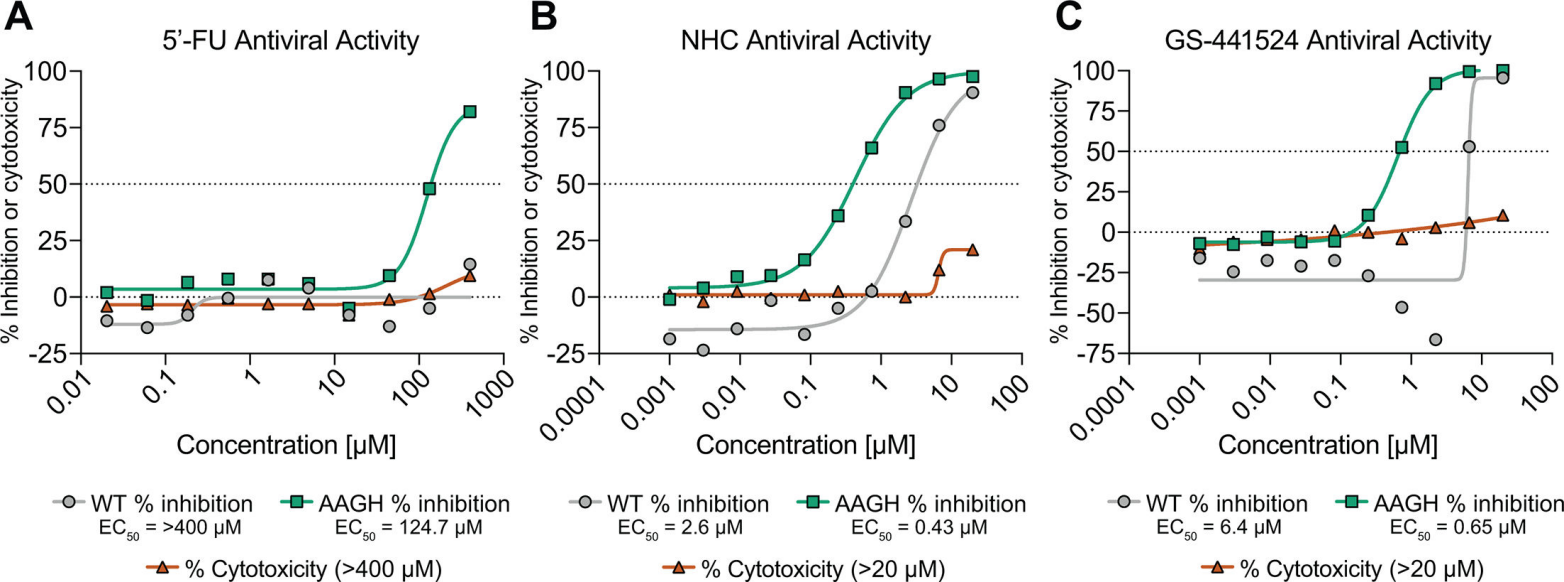
SARS-CoV-2 AAGH virus has increased sensitivity to nucleoside analogs. VTA cells were infected with nanoluciferase-expressing SARS-CoV-2 WT or AA at 37°C at an MOI of 0.1 for 1 h, after which input was removed, monolayers were washed, and then exposed to a dose response of 5-fluorouracil (5-FU) (**A**), β-d-N4-hydroxycytidine (NHC; EIDD-1931) (**B**), or GS-441524 (**C**) in infection media. Concurrently, non-infected cells were treated similarly to determine cytotoxicity. After 24 h, viral replication was assessed by NanoGlo Luciferase Assay System (Promega), and cytotoxicity was determined by CellTiterGlo Assay (Promega). Each condition was evaluated in quadruplicate in two independent studies and averaged. Values were normalized to the uninfected and infected vehicle DMSO controls (0 and 100% infection, respectively). Data were fit using a four-parameter nonlinear regression analysis using GraphPad Prism. EC50 and CC50 (cytotoxic concentration at which 50% of cells are viable) values were then determined as the concentration reducing the signal by 50%.

We have previously demonstrated that both MHV and SARS-CoV nsp14 exonuclease mutants are more sensitive to antiviral and mutagenic nucleoside analogs compared to WT viruses, functionally demonstrating the consequences of proofreading defects on replication (14, 33, 34). To determine whether SARS-CoV-2 ExoN− viruses are similarly sensitive to nucleoside analogs, we performed antiviral drug assays with SARS-CoV-2 WT and AAGH nanoluciferase reporter viruses in VTA cells (Fig. 3). We compared β-d-N4-hydroxycytidine (NHC; EIDD-1931), the parental nucleoside, to the prodrug molnupiravir; GS-441524, the parental nucleoside of the prodrug remdesivir; and 5-fluorouracil (5-FU), a known mutagenic small molecule nucleoside analog. As we had observed for MHV and SARS-CoV ExoN− viruses, SARS-CoV-2 AAGH had increased sensitivity to all small molecules assessed (14, 33, 34). Unlike WT virus, which was refractory to the effects of 5-FU even at concentrations up to 400 µM, SARS-CoV-2 AAGH was sensitive to treatment (EC_50_ = 124.7 µM) (Fig. 3A). Similarly, SARS-CoV-2 AAGH had increased sensitivity to both NHC (Fig. B) and GS-441524 (Fig. 3C) with 6- and 10-fold shifts in antiviral potency, respectively, suggesting an intrinsic role for nsp14-ExoN in the defense against antiviral nucleoside analogs (35). Thus, without a functional ExoN, SARS-CoV-2 has a notable increase in sensitivity to antiviral agents and mutagenic nucleoside analogs demonstrating the impact of ExoN-mediated proofreading on nucleoside analog sensitivity.

### SARS-CoV-2 ExoN− viruses have decreased replication fitness and fidelity

We next performed a series of studies to determine the impact of ExoN inactivation on competitive fitness and replication fidelity. We first performed head-to-head competition studies at 33°C or 37°C with SARS-CoV-2 WT and AAGH ExoN− mutant viruses in VTA cells to assess competitive fitness in low MOI infections (0.01), mixing WT or AAGH virus at infectious particle ratios of 1:1 or 1:9, the latter condition providing AAGH virus in vast excess to WT (Fig. S2A). After 18 h, supernatants were blindly passaged to naive VTA cells and then incubated for another 18 h, after which the ratios of WT and AAGH genomes present at the end of P1 and P2 were determined by Sanger sequencing (Fig. S2B). ExoN− AAGH viruses failed to compete with WT viruses at any temperature, even when introducing significantly more AAGH virus at the start of passaging (1:9, WT:AAGH), indicating that genetic inactivation of ExoN results in a significant loss in competitive fitness.

**Fig 4:**
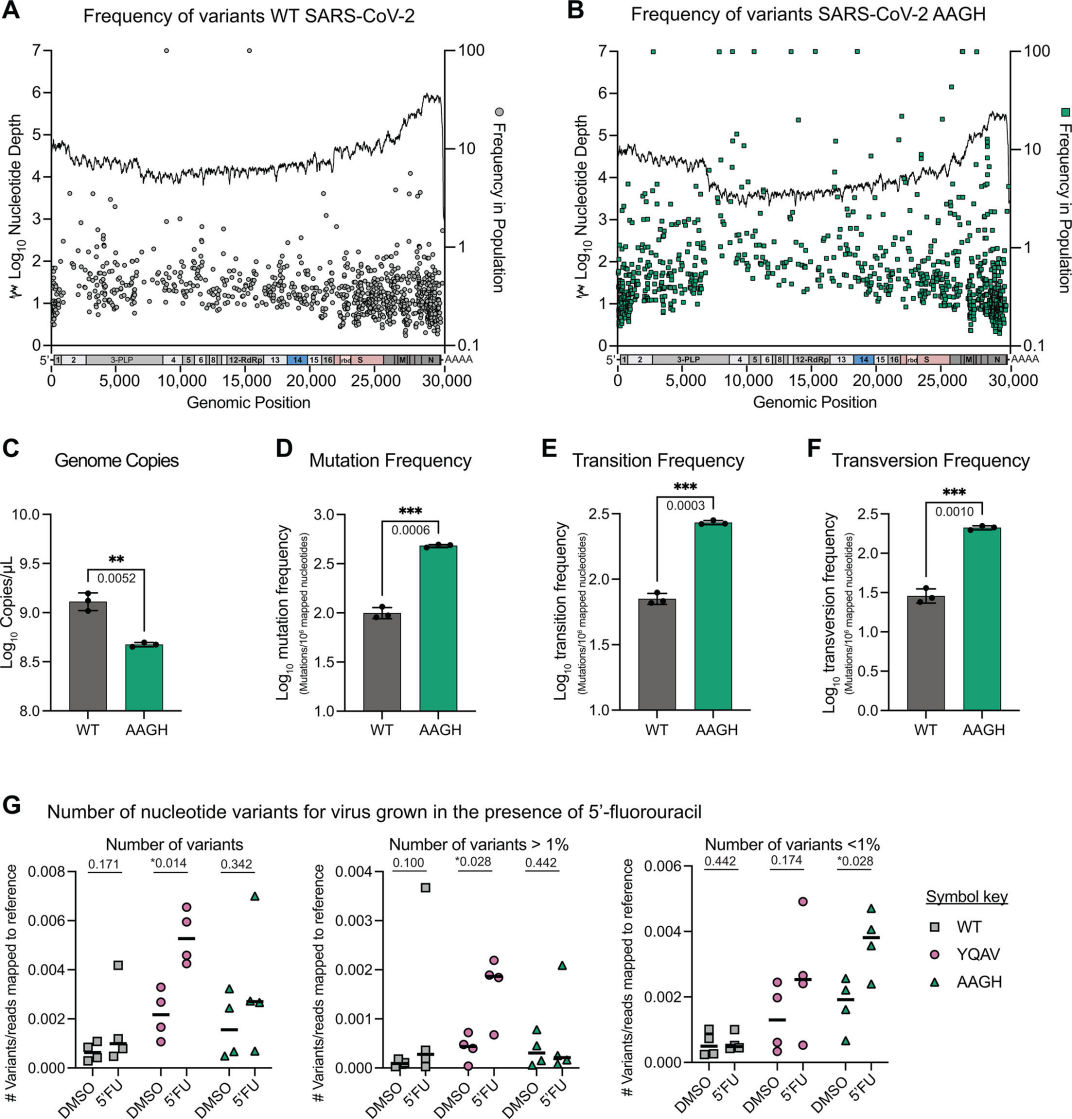
SARS-CoV-2 ExoN− viruses have decreased replication fidelity. (**A and B**) Nucleotide variant identification by deep sequencing. VTA cells were infected with SARS-CoV-2 WT or AAGH at an MOI of 0.01 in biological triplicate at 33°C until 70% of the monolayer exhibited cytopathic effect. Total RNA was isolated, and variants were identified by RNAseq. Sequence coverage, variant frequency, and location on the viral genome are shown for representative samples of WT (**A**) and AAGH (**B**) viruses. (**C**) Viral genome concentration in total RNA analyzed by RT-qPCR. (**D**) Total mutation frequency. (**E**) Transition frequency. (**F**) Transversion frequency. For panel **C**, viral genome copies were log-transformed. For panels **D–F**, the ratio of mutations per 1 million mapped nucleotides was generated and log-transformed. For panels **C–F**, the symbols represent biological replicates, the line represents the mean, error bars signify the standard deviation, and statistical significance was determined by one-tailed Welch’s *t*-test. Asterisks indicate statistical differences, and *P*-values are displayed. (**G**) Mutation frequency of WT and ExoN− viruses in the presence of 5′-fluorouracil. VTA cells were infected with SARS-CoV-2 WT, AAGH, or YQAV in the presence of DMSO vehicle or 100 µM 5′-FU at 33°C for 24 h, after which clarified supernatants were harvested, RNA extracted, and analyzed by Illumina MiSeq. Each condition was performed in quadruplicate. The total number of variants, the number of variants >1% and <1% normalized to the number of reads mapped to the reference sequence, are shown. Statistical significance determined by the Mann-Whitney test is denoted by an asterisk, and *P*-values are displayed.

The loss of nsp14 ExoN proofreading activity can manifest in an accumulation of mutations measurable by deep sequencing (12, 13, 24). Thus, we performed similar studies with select SARS-CoV-2 ExoN− loss-of-function viruses. First, we infected VTA cells with WT or SARS-CoV-2 AAGH virus at a low multiplicity of infection and harvested total RNA for Illumina RNAseq analysis when the majority of the monolayer exhibited a cytopathic effect (CPE). Sequencing read depths were similar for WT and AAGH viruses at the 5′ and 3′ ends of the genome but appeared to drop slightly for AAGH in the middle of open reading frame 1a (ORF1a) through to the M gene (Fig. 4A and B). Nevertheless, 99.9% of the AAGH genome had a sequencing coverage greater than 1,000×. An increased number of variants were detected in AAGH virus samples as compared to WT virus (Fig. 4A and B). Although AAGH sequencing samples had fewer viral genomes than WT (Fig. 4C), the mutation frequency of AAGH virus was significantly increased compared to WT virus (Fig. 4D) with notable increases in both transition and transversion mutations (Fig. 4E and F). Similar data were gained when similarly evaluating our panel of motif-I mutants and related WT SARS-CoV-2 MA10 (Fig. S3 and S4). We next sought to determine whether ExoN− virus grown in the presence of the mutagen 5-FU would result in an accumulation of variant mutations as we had previously shown with SARS-CoV (14). We infected VTA cells with WT or SARS-CoV-2 AAGH or YQAV viruses in the presence of DMSO vehicle or 100 µM 5-FU. After 24 h, viral RNA was isolated from clarified supernatants for deep sequencing analysis. SARS-CoV-2 ExoN− viruses grown in the presence of a mutagenic small molecule 5-FU accumulate mutations at a greater rate (Fig. 4G) than the same viruses grown in media containing vehicle, thus demonstrating the functional consequence of diminished replication fidelity and proofreading activity.

### SARS-CoV-2 ExoN mediates viral RNA recombination

**Fig 5:**
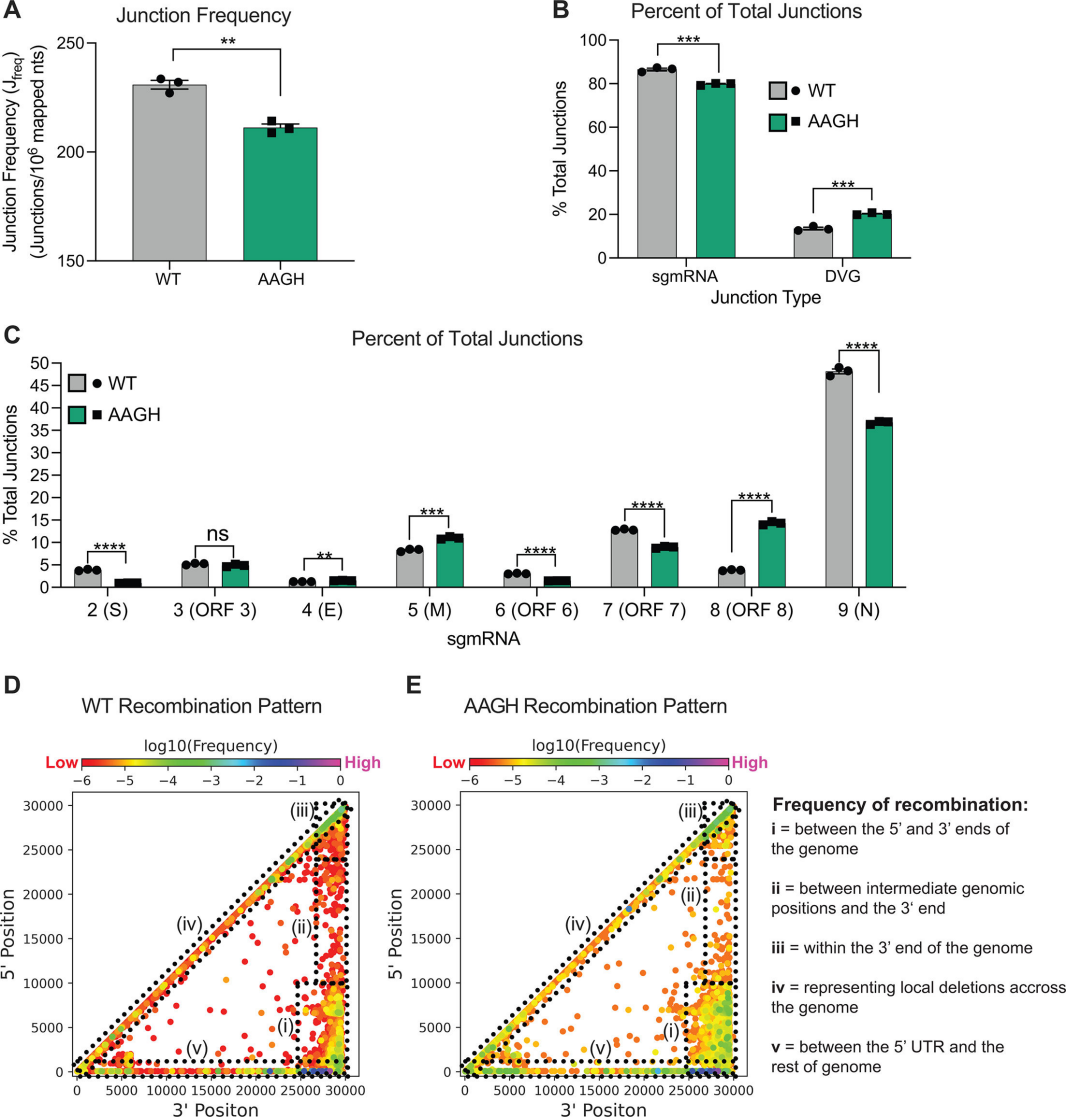
SARS-CoV-2 ExoN mediates viral RNA recombination. VTA-infected monolayer RNAs from experiments shown in Fig. 4 were analyzed by RNAseq and ViReMA. (**A**) The junction frequency (JFreq) was calculated as the ratio of detected junctions per 1 million mapped nucleotides. (**B**) Junction frequencies were calculated for DVGs and total subgenomic RNAs (sgmRNA) and plotted as the percentage of total mapped junctions. (**C**) Individual sgmRNA junction frequencies from panel B are shown as the percentage of total mapped junctions. Graphed are individual values, mean, and ± SEM error bars from three independent experiments, *N* = 3. **P < 0.01, ***P < 0.001, ****P < 0.0001, ns = not significant as determined by unpaired *t*-test. WT (**D**) and AAGH (**E**) recombination junctions are mapped according to their genomic position (5′ junction site starting position, 3′ junction site stop position) and colored according to their frequency in the population of all mapped junctions. The highest frequencies are purple, and the lowest frequencies are red. Dashed boxes represent clusters of junctions (i) 5′ → 3′, (ii) mid genome → 3′, (iii) 3′ → 3′, (iv) local deletions, (v) 5′ UTR → rest of genome. Shown are representative results from one of three independent experiments with similar outcomes.

CoV structural and accessory ORF proteins are translated from a nested set of subgenomic viral RNAs (sgRNA) that all share the same 5′ leader sequence with genomic RNA (36). CoV sgRNA acquires the distal 5′ leader sequence through a process called discontinuous transcription, a high-frequency copy-choice RNA recombination-like process where intramolecular recombinant viral mRNAs are generated (36, 37). Aside from CoV sgRNA transcription, viral recombination can also generate defective viral genomes (DVGs) of unclear function with intact 5′ and 3′ untranslated regions (UTR) and various genomic region deletions (38). To determine whether genetic inactivation of SARS-CoV-2 nsp14 ExoN would alter the patterns of viral RNA recombination, we analyzed our WT or SARS-CoV-2 AAGH deep sequencing data from Fig. 4 using ViReMa (Virus Recombination Mapper), a recombination-aware mapper (39). Junction frequency (Jfreq; the total number of junction nucleotides per million mapped nucleotides) was diminished in SARS-CoV-2 AAGH viral RNA (Fig. 5A). Of the total junctions mapped, SARS-CoV-2 AAGH had significantly fewer canonical sgmRNA junctions compared to WT, with a proportional increase in DVG junctions compared to WT (Fig. 5B). SARS-CoV-2 AAGH infection resulted in significantly fewer Spike, ORF 6, ORF 7, and N junctions, and significantly higher E, M, and ORF 8 junctions (Fig. 5C). To better understand whether recombination was enriched in specific regions of the genome, we mapped forward (5′ to 3′) recombination junctions according to their genomic position. With SARS-CoV-2 AAGH, we observed fewer junctions within the 3′ end of the genome (i.e., region iii) and between intermediate genomic positions and the 3′ end (i.e., region ii) but more junctions between the 5′ and 3′ ends (i.e., region v) compared to WT (Fig. 5D). Similar analysis was performed with our panel of motif-I mutants and related WT SARS-CoV-2 MA10 (Fig. S5). Like specific infectivity, the impact on viral RNA recombination varied with ExoN motif-I mutation. Interestingly, the RAYF mutant, which had an S.I. most similar to AAGH, had a recombination pattern most like AAGH with diminished Jfreq and sgmRNA junctions, an increase in DVGs, and a similar pattern when recombination was mapped to genomic position (Fig. S5). These data are consistent with our previous reports with MHV nsp14-ExoN− virus (38). Altogether, we demonstrate that SARS-CoV-2 nsp14-ExoN is key to replication fidelity, proofreading, and RNA recombination.

### SARS-CoV-2 ExoN− viruses are attenuated *in vivo*

**Fig 6:**
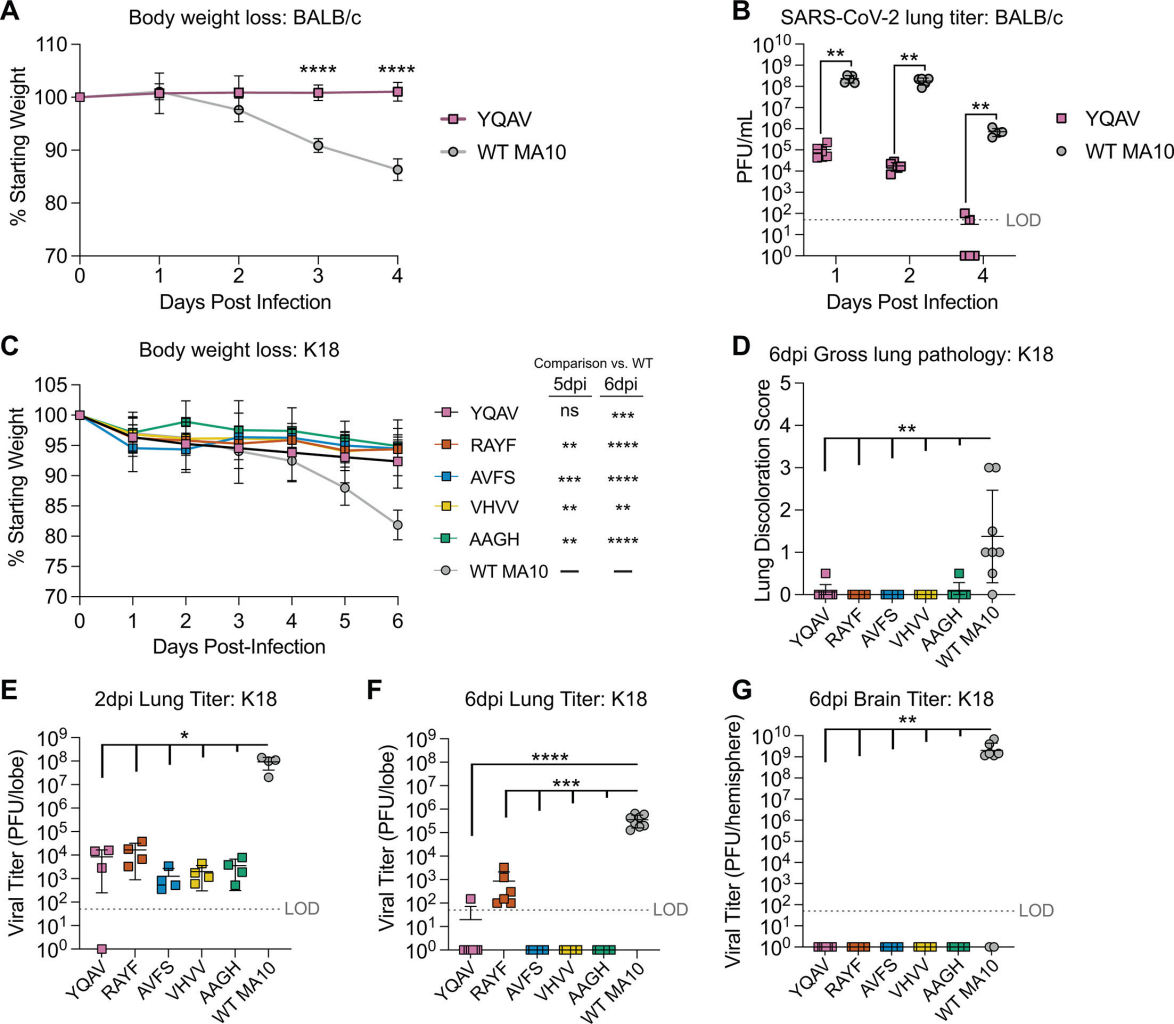
SARS-CoV-2 ExoN− viruses are attenuated in multiple mouse models. (**A**) Body weight loss in BALB/c mice. Ten-week-old female BALB/c mice were intranasally infected with 6.8E+05 PFU of WT SARS-CoV-2 MA10 (*N* = 15) or related YQAV virus (*N* = 15), and body weight was measured daily. (**B**) Viral lung titer in BALB/c mice. On the indicated day, the bottom right lung lobe was harvested from animals described in panel A and SARS-CoV-2 titers were determined on VTA cells by plaque assay. LOD = limit of detection. (**C**) Body weight loss in C67BL/6 K18 hACE2 mice. Eight- to nine-month-old male and female C57BL/6 K18-hACE2 mice were infected with 3.0E+04 PFU of WT SARS-CoV-2 MA10 (*N* = 12) or related ExoN− viruses AAGH (*N* = 12), RAYF (*N* = 10), AVFS (*N* = 10), VHVV (*N* = 10), or YQAV (*N* = 12), after which body weight was measured daily. (**D**) Gross lung pathology. SARS-CoV-2 infection can cause a hemorrhage-like lung discoloration scored on a scale of 0 (normal) to 4 (100% discolored). (**E–G**) Viral titers in the lung on 2 dpi (**E**) and 6 dpi (**F**) or brain on 6 dpi (**G**). For the longitudinal body weight data, statistical significance was determined using a mixed-effects analysis model with multiple comparisons. For the lung hemorrhage score and lung and brain titer data, statistical significance was determined via a one-tailed Mann-Whitney test. Asterisks indicate statistical differences (ns = not significant, *P value < 0.05, **P value < 0.01, ***P value < 0.001, ****P value < 0.0001). Panels **A and B** and panels **C–G** result from single independent studies.

We next evaluated the replicative fitness and pathogenic potential of SARS-CoV-2 ExoN− viruses in animal models. Since we generated ExoN mutant viruses in the mouse-adapted MA10 genetic background, we first evaluated pathogenesis in 10-week-old BALB/c mice infected with 6E+05 PFU SARS-CoV-2 MA10 WT or SARS-CoV-2 MA10 YQAV virus. The YQAV mutant was chosen for these studies because the stock titers for other mutants were not high enough to match the 6E+05 of WT. Unlike WT virus, which caused progressive body weight loss over time, SARS-CoV-2 MA10 YQAV infection did not cause weight loss during this study (Fig. 6A). Concordant with the inability to cause body weight loss, SARS-CoV-2 MA10 YQAV replication in lung tissue was significantly decreased by three to four logs, as compared to WT virus titers on 1, 2, and 4 days post-infection (dpi) (Fig. 6B). Since SARS-CoV-2 MA10 YQAV was not pathogenic in BALB/c mice, we then sought to evaluate ExoN− virus pathogenesis in a highly susceptible mouse model, C57BL/6 mice with hACE2 expressed via the epithelial K18 promoter (“K18” mice). In addition, we sought to evaluate our entire panel of ExoN− SARS-CoV-2 to more comprehensively assess pathogenic potential. We infected 8- to 9-month-old male and female K18 mice with ~3E+04 PFU of SARS-CoV-2 MA10 ExoN− panel viruses. Similar to infection in BALB/c, WT virus infection caused progressive weight loss in K18 mice, yet ExoN− viruses infection failed to induce body weight loss (Fig. 6C). Gross lung pathology was evident in only WT infected mice on 6 dpi (Fig. 6D). ExoN− virus replication in the lung at both 2 and 6 dpi was significantly attenuated as compared to WT virus in K18 mice, with mean titers differing by approximately 4 logs (Fig. 6E and F). Of ExoN− viruses, only RAYF had measurable virus in the lung on 6 dpi suggesting there may be subtle differences in replicative fitness *in vivo* among the panel viruses (Fig. 6F). Lastly, mean brain titers differed by approximately 9 logs, with evident neuroinvasion and replication in most WT-infected animals, while the brain titers of all ExoN− viruses were below the limit of detection (LOD; Fig. 6G). Altogether, these data indicate that SARS-CoV-2 MA10 ExoN− viruses are severely attenuated for replication and pathogenesis in WT mice and in the highly susceptible K18 hACE2 mouse model.

### Interferon deficiency partially restores SARS-CoV-2 ExoN− replicative fitness *in vivo*

**Fig 7:**
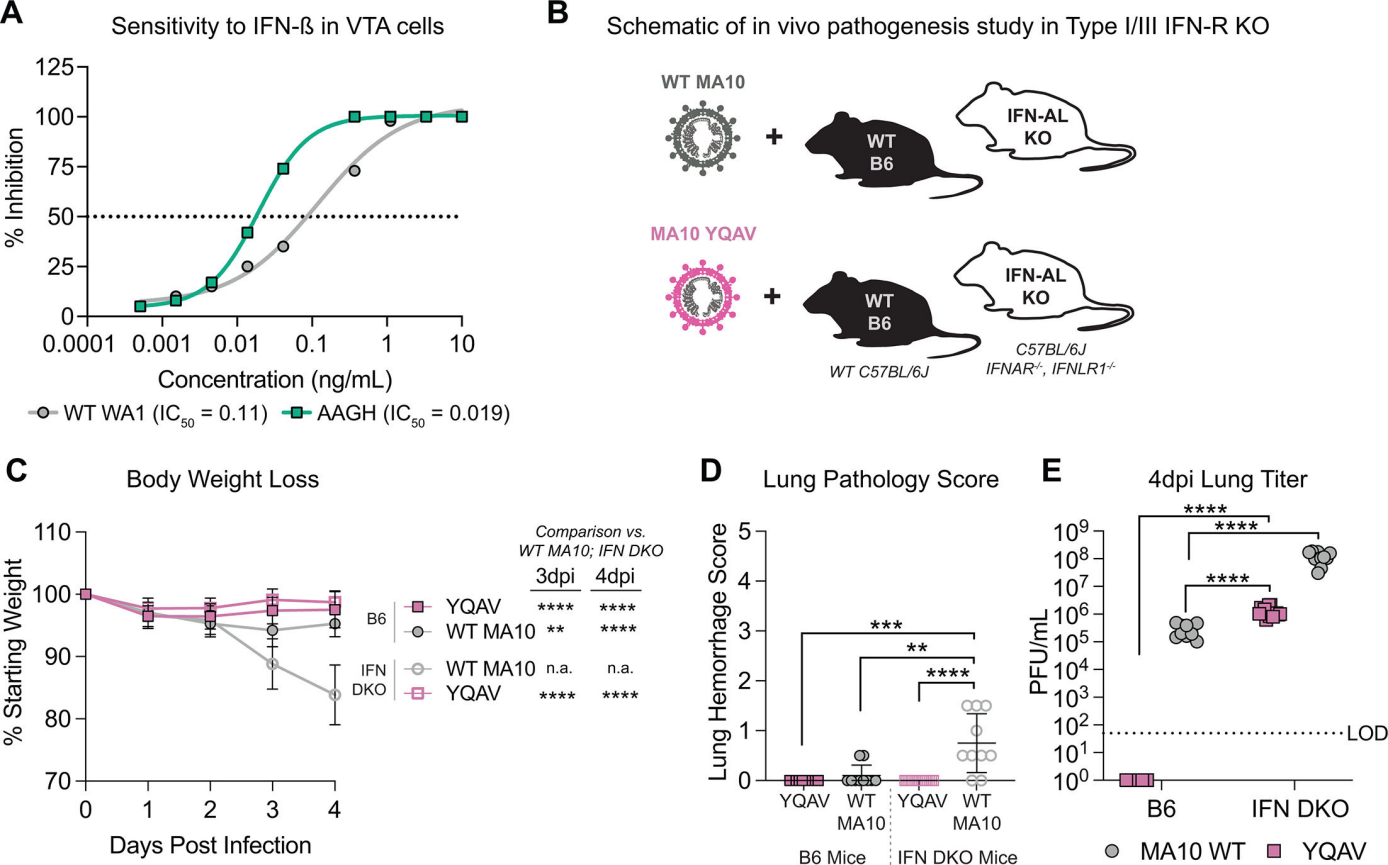
Interferon deficiency partially restores SARS-CoV-2 ExoN− replicative fitness *in vivo*. (**A**) Sensitivity to interferon beta in VTA cells. VTA cells were pre-treated with a dose response of IFNβ for 24 h. Interferon was removed, and then cells were infected with SARS-CoV-2 WT or AAGH nanoluciferase-expressing viruses in quadruplicate at an MOI of 0.1. The plates were incubated at 37°C for 1 h, the input virus was removed, cultures were washed, and growth medium was added. After 72 h at 37°C, viral replication was mediated by NanoGlo Assay. Data were generated from a single study. (**B**) Schematic of the type I and III receptor KO mouse experiment. Male and female WT mice (8 to 15 weeks old) or interferon αβλ receptor-deficient mice were infected with 6.8E+05 PFU of either WT SARS-CoV-2 MA10 (WT *N* = 10, KO *N* = 11) or YQAV virus (WT *N* = 10, KO = 15). (**C**) Body weight loss over time in mice is described in panel **B**. (**D**) Gross lung pathology score for mice described in panel **B**. (**E**) Viral lung titer 4 dpi via plaque assay. LOD = limit of detection. Asterisks indicate statistical significance by two-way ANOVA with a Dunnett’s multiple comparison test in panel **C** and a two-way ANOVA with a Tukey’s multiple comparison test in panels **C** and **D**. Data were generated from a single study.

To determine whether IFN signaling was playing a role in the attenuation of SARS-CoV-2 ExoN− replication in mice, we first assessed SARS-CoV-2 ExoN− virus susceptibility to IFN pretreatment *in vitro* (Fig. 7A) (40). The potency of IFN-β in VTA cells was 10-fold greater against SARS-CoV-2 AAGH virus (IC_50_ = 0.019 ng/mL) compared to WT virus (IC_50_ = 0.11 ng/mL), thus confirming the increased IFN sensitivity of SARS-CoV-2 ExoN− viruses. We found that SARS-CoV engineered to have the exact same ExoN−inactivating AAGH mutation also exhibited increased sensitivity to IFN-β (Fig. S6) (24).

To elucidate the importance of IFN signaling in replicative fitness and pathogenic potential *in vivo*, we infected WT mice or those deficient in type I (alpha/beta [α/β]) and type III (lambda [λ]) IFN receptor (IFN-AL KO) with either WT SARS-CoV-2 MA10 or SARS-CoV-2 MA10 YQAV virus (Fig. 7B). Like the above studies, WT infection caused progressive body weight loss, yet this metric was not affected by ExoN− virus infection (Fig. 7C). Only WT infection caused measurable gross lung pathology on 4 dpi (Fig. 7D). While SARS-CoV-2 MA10 YQAV infectious titers in WT mice were below the LOD on 4 dpi, remarkably, they were approximately 4.5 logs above the LOD in KO mice and exceeded that achieved by WT virus in WT mice at similar times (Fig. 7E). WT viral replication increased in type I/III IFN-R KO mice as well, although only by approximately 3 logs (Fig. 7E). We next performed similar studies with SARS-CoV MA15 WT and ExoN− virus (i.e., AAGH) in WT mice and those deficient in type I and type II IFN signaling (IFN-AG KO) (Fig. S6B). As expected, WT SARS-CoV MA15 virus infection in either strain of mouse caused progressive body weight loss, with mice meeting humane endpoints for euthanasia by 4 dpi (Fig. S6C). Although weight loss and clinical disease were significantly attenuated in SARS-CoV MA15 AAGH-infected WT mice, increased weight loss was observed in type I/II IFN-R-deficient mice, indicating a partial restoration of pathogenesis. Early in infection, SARS-CoV MA15 AAGH viral replication was decreased ~2 log in the lungs of either mouse strain compared to WT virus (Fig. S6D). At 4 dpi, viral replication for both WT and SARS-CoV MA15 AAGH was increased ~2 log in type I/II IFN-R-deficient mice compared to WT. Altogether, these studies indicate that SARS-CoV-2 ExoN− viruses do not have a generalizable defect in replication that limits replicative capacity *in vivo*. In addition, these data demonstrate that IFN signaling plays a significant role in attenuating replication and pathogenesis of both SARS-CoV and SARS-CoV-2 ExoN−, suggesting that ExoN function in virus and host innate immune interactions is important for replicative fitness and pathogenesis *in vivo*.

## DISCUSSION

CoV replication and transcription are coordinated by the RdRp and a variety of viral non-structural proteins that make up the RTC. While other RNA viruses, like Lassa virus, encode proteins with exonuclease activity (41), CoV exonuclease (ExoN, nsp14) uniquely exhibits proofreading function that enhances replication fidelity and is thought to facilitate the maintenance of their relatively large RNA genomes (9, 12–14). Although reverse genetic approaches to study CoV lacking ExoN enzymatic activity were successful for SARS-CoV and MHV (10, 12, 13), similar approaches with *Alphacoronavirus*, TGEV, and HCoV-229E, or Beta-CoV, MERS-CoV, and SARS-CoV-2, have been reported to fail, suggesting there may be differences in ExoN function across the CoV family (10, 26, 42). Coupling reverse genetics, saturation mutagenesis, and specific culture conditions, we recovered multiple SARS-CoV-2 mutants with mutations in ExoN motif I, including the historical D90A/E92A (AAGH) inactivating mutations. Of the mutants recovered, none grew better than the WT virus. For recovery, we utilized two cell lines with known defects in type I IFN signaling: the highly transfectable baby hamster kidney cells (BHK) for electroporation and the highly permissive Vero E6 cell overexpressing key viral entry factors, ACE2 and TMPRSS2 (i.e., VTA cells), for co-culture with BHK (43, 44). In addition, we utilized a lower temperature (33°C) for recombinant recovery, surmising that lower temperature may slow cell division, alter viral protein structure/function, and/or diminish innate immune function, the latter of which has been shown for chikungunya virus and rhinovirus (45, 46). Once recovered, SARS-CoV-2 ExoN−viruses grew similarly at 33°C and 37°C, indicating a lack of temperature-sensitive growth and that lower temperature more likely potentiates recovery by other mechanisms. Altogether, this new approach expands the viral genetic tools available for the study of CoV, enabling the investigation of SARS-CoV-2 ExoN function in replication and pathogenesis and potentially facilitating the rescue of other previously non-recoverable mutant viruses (24, 47).

The evolution of the CoV nsp14-ExoN suggests it provides a survival benefit. Although the genomes of SARS-CoV and SARS-CoV-2 are approximately 80% identical, their nsp14 proteins are 95% identical (48). Despite these similarities, there are some notable phenotypic differences among SARS-CoV and SARS-CoV-2 ExoN− viruses. SARS-CoV-2 ExoN− is more attenuated for replication in Vero cells than SARS-CoV ExoN, whose replication mirrors that of the WT virus for the first 24 h of infection (24). SARS-CoV-2 ExoN− virus is also more sensitive to interferon pretreatment than SARS-CoV ExoN. Although performed in different cells, MHV ExoN− virus growth in murine DBT cells is significantly attenuated, more akin to SARS-CoV-2 ExoN− described herein (12). These differences aside, ExoN-deficient SARS-CoV, SARS-CoV-2, and MHV all exhibit a similar loss of replication fidelity, proofreading, and specific infectivity (12–14, 24, 33). CoV subgenomic RNA transcription is mediated by a highly regulated process called discontinuous transcription, where the RTC pauses during minus-strand synthesis and switches its template to a region of sequence homology at the 5′ end of the genome template to complete the synthesis, resulting in an intramolecular recombinant (36, 49). Thus, discontinuous transcription is akin to similarity-assisted copy-choice RNA recombination, where the “donor” and “acceptor” regions of sequence identity are the transcriptional regulatory sequences (TRS) upstream of each subgenomic ORF and the TRS in the 5′ leader of the genome (36, 37). During co-infection with disparate CoVs, this same process can drive genetic shift through the exchange of viral genetic elements, which can result in host range expansion, especially when exchanging spike gene sequences (50, 51). Interestingly, SARS-CoV-2 endonuclease nsp15 has been shown to regulate viral RNA recombination (52). Here, we provide evidence that SARS-CoV-2 ExoN mediates viral RNA recombination during replication and transcription, like that of MHV, although the molecular mechanisms remain unclear (38). Future nsp14 ExoN structure and function studies may help elucidate ExoN function in CoV viral RNA recombination. The constellations of mutations recovered in our saturation mutagenesis studies may provide new insights into nsp14 ExoN function. We recovered multiple viable viruses with a variety of substitutions at nsp14 residues D90, E92, G93, and H95, where D90 and E92 are motif I active site residues and G93 and H95 interact with 3′ end nucleotides in the product strand (23). Simultaneous changes to all four amino acid residues noted above thus represent potentially significant structural and mechanistic alterations to nsp14-ExoN activity. The fact that single mutants at either D90 or E92 were not detected after passage of our saturation mutagenesis virus library and that mutation D90 or E92 was associated with additional changes in motif-I suggests that this region may have functions beyond canonical exonuclease activity. It is especially interesting that charged residues were enriched in motif I of ExoN when absent in the typical D90/E92 positions. Given that nsp14 plays a central role in the multi-protein RTC complex and engages nsp10 and 16, the changes introduced by saturation mutagenesis may impact other important RTC functions aside from inactivating nsp14-ExoN function (11, 53). Thus, the attenuation of SARS-CoV-2 ExoN− replication may in part be driven by a combination of ExoN intrinsic effects on replication fidelity and extrinsic effects on viral RTC protein interactions and functions.

The majority of the CoV nsp14 host response literature relates to its C-terminal N7-Mtase activity, which plays an essential role in generating a proper 5′ RNA cap structure, without which viral RNAs would be ripe targets for pattern recognition and induction of innate immunity (54, 55). Only a handful of studies have suggested a link between nsp14-ExoN and the innate immune response. MHV ExoN− virus is attenuated for replication and has increased sensitivity to IFN pretreatment in culture, but with passage, compensatory mutations restore replicative fitness without reversion of ExoN, and IFN sensitivity remains (40, 47). These studies suggest a function for ExoN 3′−5′ exonuclease activity in IFN antagonism. A recent overexpression study demonstrated that both nsp14 ExoN and N7-Mtase domains of SARS-CoV-2 diminish IFN-stimulated gene (ISG) translation, suggesting that nsp14 may have direct host modulatory activities (56). It is important to note that these studies were done in the absence of viral RNA and viral replication (56). Genetically unrelated Lassa virus NP protein exonuclease digests viral double-stranded RNA, thereby diminishing viral molecular patterns that can induce the innate antiviral response (41, 57). If CoV ExoN similarly antagonizes the host response, this function would not be detected in overexpression studies in the absence of viral RNA synthesis. Here, we show that SARS-CoV and SARS-CoV-2 ExoN− viruses are more sensitive to IFN pretreatment *in vitro* and are significantly attenuated in WT mice, but attenuation *in vivo* can partially be restored via knockout of IFN signaling. These data suggest that SARS-CoV-2 ExoN− attenuation is not simply due to gross defects in replication but rather signals the potential for conserved CoV ExoN functions in direct and/or indirect innate immune antagonism. Given the literature noted above, CoV ExoN may reduce the proportion of defective viral particles or double-stranded viral RNA, thereby reducing agonists of the innate immune system while also potentially directly interfering with ISG protein production. Future studies are focused on uncoupling the innate antagonist and replication fidelity phenotypes of SARS-CoV-2 ExoN− virus *in vitro and in vivo*.

Collectively, these data argue for an expansion of CoV nsp14-ExoN function. In summary, we demonstrate that CoV ExoN is not only a key mediator of replicative fitness and fidelity but is also important for subverting the innate immune response. Like our prior work with MHV and SARS-CoV, we show that SARS-CoV-2 ExoN− LOF viruses have diminished replication fidelity and competitive fitness, have increased sensitivity to nucleoside analog antivirals, have altered recombination patterns, are attenuated in primary human airway epithelial cells, and are attenuated for replication and pathogenesis in typical laboratory mouse models. However, replicative fitness *in vivo* is partially restored in the absence of interferon signaling, indicating that *in vivo* attenuation in WT mice is not driven by gross defects in replication. These data suggest that the CoV replicase is not just a governor of viral RNA synthesis, but that nsp14-ExoN mediates virus and host interactions that are essential to subvert the host antiviral response. Together, these data reveal new insights into the complexities of CoV replication and virus and host interactions, which could be leveraged for the development of novel multifaceted therapeutics that attack the ever-expanding functions of the CoV RTC in replication and pathogenesis.

## MATERIALS AND METHODS

### Cells and viruses

#### Cells

Vero E6 cells were obtained from the United States Army Medical Research Institute of Infectious Diseases (USAMRIID) and cultured in Dulbecco’s modified Eagle medium (DMEM) (Gibco) supplemented with 10% fetal bovine serum (FBS) (Gibco), and 100 U/mL penicillin (Gibco) (Complete DMEM). Vero E6 cells overexpressing the human transmembrane protease, serine 2 (TMPRSS2), and human ACE2 receptor (VTA cells) were gifted from A. Creanga and B. Graham, National Institutes of Health (NIH), and grown in DMEM supplemented with 10% FBS and passaged in the presence of 10 µg/mL puromycin dihydrochloride (Corning). Cells were routinely washed in Dulbecco’s PBS without calcium chloride or magnesium chloride (PBS −/−). Cells were maintained at 37°C, 5% CO_2_, and detached during passage and expansion with 0.05% trypsin-EDTA (Gibco). BHK-21 (ATCC CCL-10) cells were propagated in DMEM (Gibco) supplemented with 10% FBS (Gibco), penicillin-streptomycin (Gibco), and L-glutamine (Gibco). MA-104 cells (ATCC CRL-2378.1) were grown in EMEM (Gibco) supplemented with 10% FBS (Gibco), penicillin-streptomycin (Gibco). Cell growth conditions during experimentation are listed in context.

#### Viruses

All infectious clone SARS-CoV-2 were based on the WA1 isolate (WT: GenBank MT461669.1; nLuc: GenBank MT844089; MA10: GenBank MT952602) (27, 30). Wild type (WT) infectious clone SARS-CoV-2 used for this study was recovered using the reverse genetics system described previously (27, 30), in Vero E6 cells at 37°C, P0 stocks. P1 stocks were generated in VTA cells at either 33°C or 37°C and used in the experiments described. SARS-CoV-2 WA1 and MA10 ExoN mutants (i.e., YQAV, RAYF, AVFS, VHVV, and AAGH) were recovered through electroporation of BHK cells and coculture with VTA cells at 33°C. SARS-CoV-2 ExoN viruses were passaged twice to obtain working stocks for the studies described herein. SARS-CoV MA15 and SARS-CoV MA15 AAGH were generated as previously described (24). All working stocks were deep sequenced to identify mutations acquired during amplification. See Tables S2 and S3 for stock sequencing information.

### Saturation mutagenesis and recovery of SARS-CoV-2 nsp14-ExoN motif-I viruses

SARS-CoV-2 nsp14-ExoN motif-I mutant libraries were engineered through saturation mutagenesis on amino acid residues 90, 92, 93, and 95 of SARS-CoV-2 nsp14-ExoN motif-I based on previously published protocols (58, 59). The SARS-CoV-2 WA1 and MA15 infectious cDNA genomes are split up among seven plasmids (A–G) (27, 30). The MA10 genome has seven amino acid changes (nsp4 T285I, nsp7 K2R, nsp8 E23R, spike Q493K, Q498Y, and P499T, orf6 F7S) from WA1 (27). The E fragment of the SARS-CoV-2 WA1 cDNA clone encompasses the end of nsp13, all of nsp14, all of nsp15, and most of nsp16; thus, genetically, the E-fragment is the same for WA1 and MA10 (30). First, a “killed” E fragment was generated to eliminate the possibility of WT contamination in the cloning process. Briefly, the pUC57 SARS-CoV-2 E plasmid (6,371 bp) was digested with Bsu36I and Bpu10I, and the resultant 471 bp fragment was replaced with a gBlock (IDT) deleting 10 nt (amino acids 90–92) while also inserting a BtgZI restriction site that could be used for future PCR and the cloning of the saturation mutagenesis library. Degenerate forward NNK oligonucleotides (Integrated DNA Technologies) were used to randomly mutagenize nsp14-ExoN motif-I amino acids 90, 92, 93, and 95 while holding residue 91 constant as a valine (GTC) and residue 94 as a cysteine (SARS-CoV-2 ExoN 90, 92, 93 random + Cys (67 bases): 5′-TAA GAC ATG TAC GTG CAT GGA TTG GCT TCN NKG TCN NKN NKT GTN NKG CTA CTA GAG AAG CTG TTG G-3′) or alanine (SARS-CoV-2 ExoN 90, 92, 93 random + Ala (67 bases): 5′-TAA GAC ATG TAC GTG CAT GGA TTG GCT TCN NKG TCN NKN NKG CTN NKG CTA CTA GAG AAG CTG TTG G-3′). The above forward primers were used in PCR with reverse primer Bpu10I Rev (5′-TCTATCACATAGACAACAGGTG-3′) to generate the saturation mutagenesis mutant amplicon library. To clone the mutant amplicon library in the SARS-CoV-2 E fragment plasmid, the “killed” E fragment digested with BtgZI and Bpu10I was mixed with the mutant amplicon library by Gibson cloning using NEBuilder HiFi assembly Master Mix (NEB). Ligated DNA was concentrated, purified by the Zymo DNA Clean and Concentrator-5 kit, electroporated into DH10B ElectroMax cells (Invitrogen), and directly plated on multiple 5,245 mm^2^ bioassay dishes (Corning) to avoid bias from bacterial suspension cultures. Transformants were pooled and purified using a Maxiprep kit (Qiagen). The resultant E fragment library was then digested and utilized in a ligation reaction with the remaining viral cDNA fragments (A–D, F–G) to generate full-length SARS-CoV-2 MA10 viral cDNA. This cDNA was used as a template for *in vitro* transcription. The in vitro-transcribed SARS-CoV-2 RNA library was electroporated in BHK cells and cocultured with VTA cells, and the viral supernatants were passaged twice every 4–5 days in VTA cells for the enrichment of infectious genomes. RNA was isolated from supernatants with TRIzol LS. Purified RNA was prepared for sequencing using the Illumina RNA Prep with Enrichment (Illumina, 20040536) workflow, following the instructions for the Respiratory Virus Oligo Panel (Illumina, 20044311). The resulting libraries were run on a MiSeq instrument, with at least 1.5 million reads per sample. Sequences were analyzed and displayed using custom Perl, Python, and R scripts as described previously (59). These scripts and usage information are available under the Saturation Mutagenesis Pipeline on the Tse lab GitHub site (https://github.com/TseLabVirology/Saturation-Mutagenesis-Pipeline/tree/main). In brief, the CAMseqv4.pl Perl script was used to extract library sequences from sequencing files. Then, these were prepared for plotting using the merge_AA.py Python script to calculate amino acid distance to the DVEGCH wild-type sequence. The resulting data were input into R, enrichment scores were calculated, and plotted using bubbpleplot_enriched_AA.r.

### Mutagenesis and recovery of D90A/E92A

SARS-CoV-2 infectious clone plasmid was used as a template for mutagenesis (30). Site-directed mutagenesis by “round-the-horn” PCR was used to generate substitutions at the indicated sites. SARS-CoV-2 E fragment was used as a template to mutate nucleotides A18,308C and T18,309A using the following primers: M1N-V4F (5′-CAGTCGAGGGGTGTCATGCTACTAGAGAAGCT-3′) and M1N-V2-5R (5′-CGAAGCCAATCCATGCACGTACATGTCTTATAGCTTC-3′), resulting in nsp14 amino acid substitution D90A. This plasmid was then used as a template to mutate nucleotides A18,314C and G18,315T using the following primers: M1N-V6F (5′-CTGGGTGTCATGCTACTAGAGAAGCTGTTGGTAC-3′) and M1N-V6R (5′-CGACTGCGAAGCCAATCCATGCAC-3′), resulting in the nsp14 double amino acid substitution D90A/E92A. All primers were 5′-phosphorylated with T4 polynucleotide kinase using an ATP-containing reaction buffer (NEB). Template backbone DNA was digested with DpnI (NEB), and amplified DNA was separated by electrophoresis and extracted from agarose (Promega). Ligated DNA was transformed into top 10 competent *Escherichia coli* cells (Thermo) and amplified in liquid culture, and sequences were confirmed by Sanger sequencing. Assembly and recovery of recombinant SARS-CoV-2 have been described previously (30), with the following modifications. VTA cells were electroporated with *in vitro* transcribed, full-length genome assemblies and grown at 33°C 5%CO_2_. When the cytopathic effect consumed approximately 80% of the monolayer, clarified supernatants were aliquoted and stored at −80°C (passage 0 [P0]). The P0 stocks were then passaged in VTA cells at either 33°C or 37°C, P1. P1 stocks of 33°C or 37°C grown virus cultures were used for most *in vitro* experiments presented. Engineered mutations of the P0 or P1 stocks were confirmed by Sanger sequencing of PCR amplicons harboring the sequence of interest (each 3–4 kb in length).

### Replication kinetics of SARS-CoV-2 ExoN− viruses in VTA cells

VTA cells (4E+05) were seeded per well per six-well plate the previous day to generate sub-confluent monolayers. The VTA cells were infected in triplicate with the SARS-CoV-2 MA10 WT or related ExoN mutant (i.e., AAGH, RAYF, AVFS, VHVV, or YQAV) viruses at an MOI of 0.01 in infection medium (DMEM, 5% FBS, 1× Pen/Strep) for 1 h at 37°C with rocking. After infection, the virus was removed, the monolayers were washed with 2 mL PBS without Ca^2+^/Mg^2+^, and 2 mL of infection medium was added per well. The plates were incubated at 37°C, and virus supernatants were harvested at 0 (post-wash), 8, 24, 36, and 48 h post-infection. At each timepoint, a 700 µL sample of the infection supernatant was collected, clarified (13,000 rpm, 5 min), and stored at −80°C. 700 µL of fresh, warmed infection medium was added back to each well at each collection timepoint. The virus supernatants were titered by plaque assay in VTA cells as described previously (30).

### Virus replication assays to determine specific infectivity

VTA cells were plated at a density of 4E5 cells per well in six-well plates 24 h before infection. Cells were then infected at an MOI of 0.01 PFU/cell and incubated at either 33°C or 37°C for 30 min. Inocula were removed, and the cells were washed twice with PBS containing calcium chloride and magnesium chloride (PBS +/+). Complete DMEM was added to cells, and cells were incubated at 33°C or 37°C for 48 h. At the indicated times, 700 µL supernatant samples were collected, and 700 µL of temperature-matched complete DMEM was added back. Of the 700 µL samples collected, 100 µL was added to TRIzol-LS reagent (Ambion), and the remaining 600 µL was stored at −80°C for plaque assay.

### Plaque assays and RT-qPCR

Plaque assays were performed on subconfluent VTA cells seeded in six-well plates. Serial dilutions of virus samples were plated in duplicate and overlaid with 0.9% agar in DMEM containing 5% FBS and 1× penicillin/streptomycin; cells were incubated at either 33°C or 37°C, and titers were scored 72 hpi. Genome quantification was determined by one-step RT-qPCR for supernatant and monolayer-derived RNAs extracted with TRIzol and purified with a KingFisher MagMAX Viral/Pathogen Nucleic Acid Isolation Kit (Thermo) according to the manufacturer’s protocol. Viral RNA was detected on a QuantStudio 3 real-time PCR system (Applied Biosystems) by TaqMan Fast Virus 1-Step Master Mix chemistry (Applied Biosystems) using a 5′ 6-carboxyfluorescein (FAM) and 3′ black hole quencher 1 (BHQ-1)-labeled probe (5′- ACCTACCTTGAAGGTTCTGTTAGAGTGGT-3′) and forward (5′- GTGCTCATGGATGGCTCTATTA-3′) and reverse (5′- TGTTGTCATCTCGCAAAGGCTCTCA-3′) primers corresponding to nsp4. RNA copy numbers were determined using an nsp4 RNA standard derived from the SARS-CoV-2 B fragment.

### Single-cycle infection experiments

VTA cells were plated at 1.25E5 cells per well in 12-well plates 24 h before infection. Cells were then infected at an MOI of 1.0 PFU/cell and incubated at 33°C for 30 min. Inocula were removed, and the cells were washed twice with PBS +/+. Complete DMEM was added to the cells, and the cells were incubated at 33°C for 7 h. Supernatants were removed, and cell-infected monolayers were collected in TRIzol reagent.

### Competitive fitness assay

VTA cells were plated at 4E5 cells per well in six-well plates 24 h before infection. Cells were then co-infected with three independent lineages at a total MOI of 0.01 PFU/cell at either a 1:1 ratio of WT:AAGH, MOI 0.005 PFU/cell each, or a 1:9 ratio of WT:AAGH, MOI 0.001 or 0.009 PFU/cell, respectively. Cells were incubated at either 33°C or 37°C for 30 min. Inocula were removed, and the cells were washed twice with PBS +/+. Complete DMEM, 1.5 mL, was added to cells, and cells were incubated at 33°C or 37°C for 18 h. The entire P1 supernatant sample was collected, and 50 µL was used to blindly infect VTA cells plated at the same density 24 h earlier. The entire P2 supernatant sample was collected at 18 hpi. The resulting P1 and P2 cell-infected monolayers were collected in TRIzol reagent, and viral RNA was extracted by chloroform extraction and purified using the KingFisher MagMAX Viral/Pathogen Nucleic Acid Isolation Kit. Viral cDNA was generated with SuperScript IV reverse transcriptase using random hexamers and oligo(dTs). Amplicons were generated via PCR using EasyA polymerase and Sanger sequenced. Sanger sequencing traces at genome positions 18,308, 18,309, 18,314, and 18,315 were then analyzed by area under the curve, and the pooled percentage of either wild type or engineered mutant nucleotide was graphed.

### SARS-CoV-2 nucleoside analog sensitivity studies

VTA cells (2E+04) were seeded in 100 µL growth medium (DMEM, 10% FBS, 1× NEAA, 1× Pen/Strep, 10 µg/mL puromycin) per well in a black-bottomed 96-well plate the day prior to infection. For infection, medium was aspirated, and cells were infected with 100 µL of SARS-CoV-2 WT or ExoN(−) AAGH nanoluciferase-expressing viruses diluted to an MOI of ~0.1 in infection medium (DMEM, 5% FBS, 1× Pen/Strep). The plates were incubated at 37°C for 1 h. After incubation, the virus was removed, the monolayers were washed with 100 µL of infection medium, and the cells were treated with threefold serial dilution series of 5-fluorouracil (5-FU; Sigma), β-d-N4-hydroxycytidine (NHC; EIDD-1931, MedChemExpress), or GS-441524 (MedChemExpress) in infection medium, starting at concentrations of 400 µM, 20 µM, and 20 µM, respectively. The final concentration of small molecule vehicle, DMSO, was 0.2% per well. Concurrently, non-infected cells were treated with the same dose responses of the above compounds to determine cytotoxicity. The plates were incubated at 37°C for 24 h, and viral replication was determined through the measurement of nanoluciferase activity using the NanoGlo Luciferase Assay System (Promega), and cytotoxicity was determined by CellTiterGlo Assay (Promega). Each condition was evaluated in triplicate in two independent studies. Values were normalized to the uninfected and infected vehicle DMSO controls (0% and 100% infection, respectively). Data were fit using a four-parameter nonlinear regression analysis using GraphPad Prism. EC50 and CC50 (cytotoxic concentration at which 50% of cells are viable) values were then determined as the concentration reducing the signal by 50%.

### Infections for RNA sequencing and recombination analysis

VTA cells were plated at 1E6 cells per T25 flask. Cells were infected with the indicated viruses at an MOI of 0.01 and incubated at 33°C for 30 min. Inocula were removed, and the cells were washed twice with PBS +/+. Complete DMEM was added to the cells, and the cells were incubated at 33°C. Supernatants were removed, and the monolayers were collected in TRIzol when cells were ≈70% engaged in CPE, 20–45 hpi. Viral RNA was extracted by chloroform extraction and purified using the KingFisher MagMAX Viral/Pathogen Nucleic Acid Isolation Kit. Illumina RNA sequencing of viral RNA, processing, and alignment. Extracted total RNA underwent poly(A) selection followed by NovaSeq PE150 sequencing (Illumina) at 15 million reads per sample at the Vanderbilt University Medical Center core facility, Vanderbilt Technologies for Advanced Genomics (VANTAGE). VANTAGE performed base-calling and read-demultiplexing. The CoVariant pipeline was used for variant analysis (32, 38). The first module trims and aligns raw FASTQ files to the viral genome for each specified sample using a standard Bash shell script. To summarize, raw reads were processed by first removing the Illumina TruSeq adapter using Trimmomatic (60). Reads shorter than 36 bp were removed, and low-quality bases (Q score of <30) were trimmed from read ends. The raw FASTQ files were aligned to the SARS-CoV-2 genome (MT020881.1) by using the CoVariant Python 3 script and the ViReMA (Viral Recombination Mapper, version 0.21) (39) Python3 script command line parameters. For variant analysis, the sequence alignment map (SAM) file was processed using the Samtools suite (61), and alignment statistics output was generated by Samtools idxstats to an output text file. Nucleotide depth at each position was calculated from the SAM files using BBMap (Bushnell) pileup.sh.

For recombination analysis, the RecombiVIR pipeline was used (38). Following alignment, recombination junctions were filtered, quantified, and annotated by using RecombiVIR_junction_analysis.py with the following command line parameters: python RecombiVIR_junction_analysis.py samples.txt SARS2 ../directory experiment_name—version 0.21 -- Shannon Entropy ../Shannon_Entropy – Virus_Accession MT020881.1.

In summary, the recombination JFreq was calculated by comparing the number of nucleotides in detected recombination junctions to the total number of mapped nucleotides in a library. JFreq was reported as the junctions per million nucleotides sequenced. Mean JFreq values are reported. Forward recombination junctions were classified as either sgmRNA junctions or DVG junctions, based on the position of their junction sites, and filtered in module 2 of RecombiVIR (RecombiVIR_junction_analysis.py). Briefly, junction start sites were filtered to those positioned within 30 nucleotides of the transcriptional regulatory sequence leader (TRS-L) for each virus. The stop sites were then filtered for those positioned within 30 nucleotides of each respective sgmRNA TRS. This window is supported by other reports defining the flexibility of the CoV transcriptome. The JFreq of each sgmRNA was calculated by dividing the number of nucleotides in a specific sgmRNA population by the total amount of viral RNA (total mapped nucleotides). This ratio was multiplied by 10^6^ to scale, or the number of nucleotides sequenced. DVG JFreq was calculated by dividing the number of nucleotides in DVG junctions by the total amount of viral RNA in a sample (total mapped nucleotides). This ratio was multiplied by 10^6^ to scale, or the number of nucleotides sequenced. The percentage of sgmRNA and DVG junctions was then calculated by comparing the depth of all filtered sgmRNA or DVG junctions to the sum of all detected forward junctions.

### Infections for RNA sequencing of genomic RNA from DMSO or 5′ FU-treated cells

T-25 flasks of VTA cells seeded the day prior with 1E+06 cells were infected with SARS-CoV-2 WT, AAGH, or YQAV at an MOI of 0.01 for 1 h at 33°C, after which input virus was removed, monolayers were washed with PBS, and infection medium was added including DMSO vehicle or 100 µM 5′-FU. After 24 h, clarified supernatants were harvested, RNA extracted in TRIzol LS, and analyzed by Illumina MiSeq. Each condition was performed in quadruplicate. The total number of variants, the number of variants >1% and <1% normalized to the number of reads mapped to the reference sequence, are shown.

### SARS-CoV-2 nsp10/nsp14 fusion cloning protein expression

SARS-CoV-2 NSP10 fused via a 2× GGS linker to NSP14 was codon optimized and cloned into vector pK27, which includes three N-terminal tags: his tag, flag tag, and a SUMO tag. C41 (DE3) pLysS-competent cells (Sigma-Aldrich CMC0018) were transformed and grown in terrific broth at 37°C with 50 µg/mL kanamycin until the cultures reached a density between 0.8 and 1 OD_600_. Cultures were cooled down at 4°C for 1 h, induced with 0.5 mM isopropyl β-D-1-thiogalactopyranoside (IPTG), and incubated overnight on a shaker at 200 rpm and 18°C. The next day, the cultures were centrifuged at 4,000 × *g* for 10 min, and the cell pellets were resuspended in 20 mL of lysis buffer (50 mM NaH_2_PO_4_, 300 mM NaCl, 10 mM imidazole, pH 8, complete EDTA-free protease inhibitor tablet [Sigma-Aldrich 04693132001]) per 2–5 g cell pellet weight, then lysed with a cell dismembrator. Lysed cells were then centrifuged at 18,000 × *g* for 30 min at 4°C, and the supernatant was passed through a 0.45 µm filter and resuspended in a ratio of 1:5 in lysis buffer and incubated with Talon affinity resin (Takara) at 4°C for 1 h, with occasional shaking. Resin was captured via gravity chromatography, and the protein was removed with elution buffer (50 mM NaH_2_PO_4_, 300 mM NaCl, and 500 mM imidazole, pH 8), then dialyzed overnight twice against PBS to remove imidazole. Protein was then concentrated with a PES concentrator (Thermo Sci., 88541), quantitated via protein assay kit (Thermo Sci., A53225), and visualized with a 4%–20% mini-Protean TGX Stain-free gel (BioRad) imaged with a BioRad Chemidoc MP imaging system.

### FRET-based *in vitro* exonuclease assay

Exonuclease activity was evaluated using purified WT and mutant NSP10/NSP14 proteins diluted in NSP buffer [50 mM Tris-HCl, 0.0001% (vol/vol) Tween 20, 5% (vol/vol) glycerol, 1.5 mM MgCl_2_, 20 mM NaCl, 0.5 mM tris(2-carboxyethyl)phosphine (TCEP), 0.1 μg/mL bovine serum albumin (BSA)]. Proteins were left with the affinity tags intact. 500 nM WT protein was mixed with 250 nM annealed RNA oligos containing fluorophore and quencher 5′TexRd-XN/rArCrArArArArCrGrGrCrCrCrA and rA*rA*rA*rU*rA*rG*rG*rG*rC*rC*rG*rU*rU*rU*rU*rG*rU*/3′IAbRQSp/(* designates phosphothioate bond). Oligos were combined 1:1 and annealed starting at 95°C for 10 min, with a 5°C step-down every minute thereafter until a final 5-min step at 25°C. Fluorescence was measured using a plate reader (Thermo Sci., Varioskan Lux 3020-81011) at an excitation of 590 nm, and emission 615 nm for 1 h at room temperature. Interface and adaptive-mutant proteins were normalized to WT concentrations using a combination of protein quantitation assay results and protein band intensity imaged in a mini-Protean TGX Stain-Free gel.

### Evaluating the susceptibility of SARS-CoV and SARS-CoV-2 ExoN− viruses to IFNβ pretreatment *in vitro*

For SARS-CoV-2, 2E+04 VTA cells were seeded in 100 µL of growth medium (DMEM, 10% FBS, 1× NEAA, 1× Pen/Strep, 10 µg/mL puromycin) per well in a black-bottomed 96-well plate. The following day, the VTA cells were pre-treated with a threefold serial dilution series of IFNβ in quadruplicate, starting with a top concentration of 10 ng/mL, prepared in growth medium (DMEM, 10% FBS, 1× NEAA, 1× Pen/Strep, 10 µg/mL puromycin) for 24 h. The IFNβ-containing growth medium was removed, and the VTA cells were infected with 100 µL of the SARS-CoV-2 WT or ExoN(−) AAGH nanoluciferase-expressing viruses diluted to an MOI of ~0.1 in infection medium (DMEM, 5% FBS, 1× Pen/Strep). The plates were incubated at 37°C for 1 h. After incubation, the virus was removed, the monolayers were washed with 100 µL of infection medium, and 100 µL of infection medium was added to each well. The plates were incubated at 37°C, and nanoluciferase activity (used as a proxy for virus replication) was measured at 72 hpi using the NanoGlo Luciferase Assay System (Promega). For SARS-CoV, 1E+04 MA-104 cells/well in a 48-well plate were seeded 48 h prior to infection. Before 6 h of infection, cells were exposed to a dose response of IFN-β (InvivoGen): 0, 10, 100, or 500 units/mL. Cells were then infected with SARS-CoV MA15 or SARS-CoV MA15 AAGH at an MOI of 1, assuming cells have doubled. After 48 h, the levels of infectious virus in the supernatant were measured via plaque assay.

### Replication kinetics of SARS-CoV-2 ExoN− viruses in HAE cell cultures

HAE cell cultures from two independent human donors were obtained from the Tissue Procurement and Cell Culture Core Laboratory in the Marsico Lung Institute/Cystic Fibrosis Research Center at UNC. Cells were maintained in “air liquid interface” (ALI, Tissue Procurement and Cell Culture Core Laboratory) medium. Prior to infection, the apical side of each culture was washed with 0.5 mL of PBS without Ca^2+^/Mg^2+^ for 30 min at 37°C to remove excess mucus. For infection, 200 µL of SARS-CoV-2 MA10 WT or related ExoN mutant AAGH, or YQAV viruses (MOI 0.1) was added to the apical surface of HAE in triplicate and incubated 1.5 h at 37°C, after which input virus was removed, and the apical surfaces were washed with 0.5 mL Ca^2+^/Mg^2+^ for 10 min at 37°C to wash away input virus. To determine infectious virus production, the apical surfaces of cultures were washed with 200 µL PBS after 24, 48, and 72 h and titered by plaque assay in VTA cells as described above.

### Assessing replication and pathogenesis of SARS-CoV-2 ExoN− viruses in Balb/c mice

Ten-week-old female Balb/c mice were anesthetized with ketamine-xylazine and infected intranasally with 6.8E+05 PFU of WT SARS-CoV-2 MA10 (*N* = 15) or related YQAV virus (*N* = 15) diluted in PBS without Ca^2+^/Mg^2+^. Body weights were measured daily. On 1, 2, and 4 dpi, a subset of animals (*N* = 5/group) was humanely euthanized, gross lung pathology score was recorded, and the inferior right lung lobe was harvested and stored at −80°C until infectious virus titration by plaque assay in VTA cells as described previously (27). Gross lung pathology is observed with emerging CoV infection of mice, where lungs can appear “hemorrhaged.” This gross lung pathology phenotype is scored on a scale of 0–4, where 0 is a normal pink healthy lung, and 4 is a completely dark red lung.

### Assessing replication and pathogenesis of SARS-CoV-2 ExoN− viruses in K18-hACE2 mice

Eight- to nine-month-old male and female K18-hACE2 mice were anesthetized with ketamine-xylazine and infected intranasally with 3E+04 PFU WT SARS-CoV-2 MA10 (*N* = 12) or related ExoN− viruses AAGH (*N* = 12), RAYF (*N* = 10), AVFS (*N* = 10), VHVV (*N* = 10), or YQAV (*N* = 12) diluted in PBS without Ca^2+^/Mg^2+^. Body weights were measured daily. On 2 dpi, four animals per group were humanely euthanized, gross lung pathology score was recorded, and the inferior right lung lobe was harvested and stored at −80°C until infectious virus titration by plaque assay in VTA cells as described previously (27). On 6 dpi, the remaining animals were humanely euthanized, gross lung pathology score was recorded, and the inferior right lung lobe and the left brain hemisphere were harvested and stored at −80°C until infectious virus titration by plaque assay in VTA cells.

### Assessing replication and pathogenesis of SARS-CoV-2 ExoN− viruses in type I (αβ) and type III (λ) IFN receptor double knockout B6 mice

Male and female WT C57BL/6J mice (8 to 15 weeks old; Jackson Labs) and congenic C57BL/6J IFN αβλ-R KO mice (62) donated as a gift from Dr. Helen Lazear at UNC were anesthetized with ketamine-xylazine and infected intranasally with 6.8E+05 PFU of WT SARS-CoV-2 MA10 (WT; *N* = 10, KO *N* = 11) or related YQAV virus (WT; *N* = 10, KO = 15) diluted in PBS without Ca^2+^/Mg^2+^. Weights were measured daily, and on 4 dpi, animals were humanely euthanized, gross lung pathology score was recorded, and the inferior right lung lobe was harvested and stored at −80°C until infectious virus titration by plaque assay in VTA cells as described previously (27).

### Assessing replication and pathogenesis of SARS-CoV MA15 ExoN− viruses in type I (αβ) and type II (γ) IFN receptor double knockout B6 mice

Female WT C57BL/6J mice (20 weeks old; Jackson Labs) and congenic C57BL/6J IFN αβγ-R KO mice were anesthetized with ketamine-xylazine and infected intranasally with 6.8E+05 PFU of WT SARS-CoV MA15 (WT; *N* = 27, KO *N* = 25) or related SARS-CoV MA15 AAGH virus (WT; *N* = 42, KO = 41) diluted in PBS without Ca^2+^/Mg^2+^. Weights were measured daily. On 1, 2, and 4 dpi, a subset of animals from each group was humanely euthanized, and the inferior right lung lobe was harvested and stored at −80°C until infectious virus titration by plaque assay in Vero-E6 cells, as described previously (63).

### Laboratory biosafety

All *in vitro* and *in vivo* work presented herein was approved by Vanderbilt University, the UNC Institutional Biosafety Committee, and the Institutional Animal Care and Use Committee at UNC Chapel Hill. All virology was performed with approved standard operating procedures for SARS-CoV or SARS-CoV-2 in BSL3 facilities, which met requirements recommended in “Biosafety in microbiological and biomedical laboratories” by the US Department of Health and Human Services, the US Public Health Service, the US Centers for Disease Control and Prevention, and the NIH.

### Statistical analysis

GraphPad Prism, version 9 (La Jolla, CA) was used for all statistical analyses. All tests and sample sizes are listed in the figure legends.

## Data Availability

FASTQ files for the RNA sequencing variant and recombination analyses have been deposited in the National Center for Biotechnology Information Sequence Read Archive under the accession numbers PRJNA1244841 and PRJNA1245334. FASTQ files for growth of virus in the presence of small molecule treatment can be found in the Sequence Read Archive under the accession number PRJNA1374231. FASTQ files for the sequencing of saturation mutagenesis viral supernatants have been deposited in the Sequence Read Archive under the accession number PRJNA1391540.

## SUPPLEMENTAL MATERIAL

Figure S1 mbio.00073-26-s0001.pdf RNA synthesis and S.I. stocks.

Figure S2 mbio.00073-26-s0002.eps Competition assays.

Figure S3 mbio.00073-26-s0003.eps MA virus sequencing.

Figure S4 mbio.00073-26-s0004.eps MA virus mutation freqencies.

Figure S5 mbio.00073-26-s0005.eps MA virus recombination.

Figure S6 mbio.00073-26-s0006.eps SARS-CoV IFN.

Legends mbio.00073-26-s0007.docx Supplemental figure and table legends.

Table S1 mbio.00073-26-s0008.xlsx Sequences of viable saturation mutagenesis variants enriched in passage.

Table S2 mbio.00073-26-s0009.xlsx Coding mutations identified in SARS-CoV-2 AAGH virus stocks

Table S3 mbio.00073-26-s0010.xlsx Coding mutations identified in SARS-CoV-2 MA10 nsp14 active site virus stocks.

ASM does not own the copyrights to Supplemental Material that may be linked to, or accessed through, an article. The authors have granted ASM a non-exclusive, world-wide license to publish the Supplemental Material files. Please contact the corresponding author directly for reuse.
