# Supplementary material for: Loss of nsp14-exonuclease activity impairs the replication, proofreading, fitness, and pathogenesis of SARS-CoV-2
Source: mBio. 2026 May 6;17(6):e00073-26. doi: 10.1128/mbio.00073-26 (PMC13251404; doi:10.1128/mbio.00073-26)
Supplement: Figure S1 — RNA synthesis and S.I. stocks. [file mbio.00073-26-s0001.pdf]

A

Genomic RNA 7hpi with MOI 1

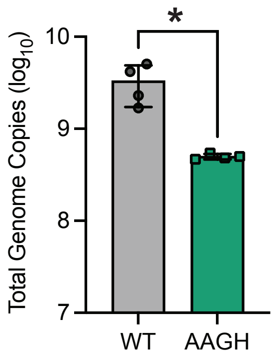

B

Specific infectivity (S.I.) of viral stocks

Virus	Genomes/mL	PFU/mL	RNA Copies/PFU	Fold Change from WT (RNA Copies/PFU)
WT	1.90E+10	4.43E+07	430	1
RAYF	6.08E+10	1.17E+06	52,024	121
AVFS	2.68E+10	8.05E+06	3,330	8
VHVV	3.35E+10	6.97E+06	4,811	11
YQAV	3.03E+10	1.37E+07	2,206	5
AAGH	4.10E+10	5.88E+05	69,756	162
