# Supplementary material for: Loss of nsp14-exonuclease activity impairs the replication, proofreading, fitness, and pathogenesis of SARS-CoV-2
Source: mBio. 2026 May 6;17(6):e00073-26. doi: 10.1128/mbio.00073-26 (PMC13251404; doi:10.1128/mbio.00073-26)
Supplement: Legends — Supplemental figure and table legends. [file mbio.00073-26-s0007.docx]

**Figure S1: SARS-CoV-2 AAGH has diminished RNA synthesis and ExoN mutant stocks have increase specific infectivity. (A)** VTA cells were infected with WT or AA at 33°C at an MOI 1.0 PFU/cell for 30 min after which input was removed and monolayers were washed twice. Total RNA from cell-infected monolayers were collected in TRIzol 7 hours post infection (hpi). RNA was purified from TRIzol, and total viral genomes were quantified by RT-qPCR. Graphed are individual values, mean, and ± SEM error bars from four independent experiments, n=4. *p<0.05 as determined by unpaired t-test. **(B)** Specific infectivity of stock viruses. Total RNA from virus stocks were collected in TRIzol. RNA was purified and viral genomes were quantified by RT-qPCR.

**Figure S2: SARS-CoV-2 AAGH virus has diminished competitive fitness. (A)** Competitive fitness study schematic. VTA cells in 6-well plates were co-infected with three independent lineages of SARS-CoV-2 WT mixed with SARS-CoV-2 AAGH at 1:1 MOI ratios of 0.005 PFU/cell or 1:9 MOI ratios of 0.001 (WT) and 0.009 (AA) PFU/cell for 1hr, input virus was removed, monolayers were washed twice and growth medium was added. After 18hr, supernatants (Passage 1, P1) were blindly passaged on VTA cells and total RNA was collected in TRizol. After 18hr, total RNA was similarly collected from P2 monolayers. RT-PCR amplicons encompassing nsp14-ExoN motif I were analyzed by Sanger sequencing. **(B)** Percent population WT or AAGH after passage. Sanger sequencing traces were analyzed for area under the curve at nucleotide positions 18,308 (D90A; WT GAT, AAGH GCA) and 18,314 (E92A; WT GAG, AAGH GCT) to determine the percent of the population of WT or ExoN- genotype. The error bars represent the mean and standard deviation. The asterisks indicate statistical significance by Mann-Whitney test.

**Figure S3: SARS-CoV-2 nsp14 active site mutants have decreased fidelity compared to WT virus.** Related to Main Figure 4. VTA cells were infected with SARS-CoV-2 MA10 WT or catalytic pocket mutants at 33˚. Viral RNA was extracted from infected cell monolayers and analyzed by RNAseq and variant calling. Coverage and variant maps for SARS-CoV-2 MA10 **(A)**, RAYF **(B)**, AVFS **(C)**, VHVV **(D)**, and YQAV **(E)**. Connected lines denote the depth of coverage, corresponding to the left y axis, and symbols denote the frequencies for the individual variants, corresponding to the right y axis. Shown are representative results from one of three independent experiments with similar outcomes.

**Figure S4: SARS-CoV-2 nsp14 active site mutants increased mutation frequency compared to WT virus.** Related to Main Figure 4 and S3. RNA and deep sequencing data was further analyzed. **(A)** Viral genome concentration determined by RT-qPCR. **(B)** Frequencies of mutations, **(C)** transitions and **(D)** transversions were determined as the ratio of mutations per 1 million mapped nucleotides. Graphed are individual values, mean, and ± SEM error bars from three independent experiments, n=3. Text insert denotes fold change compared to MA10. *p<0.05, **p<0.01, ***p<0.001, ****p<0.0001 as determined by one-way analysis of variance (ANOVA) with Dunnett’s multiple comparison test.

**Figure S5: SARS-CoV-2 nsp14 active site mutants have altered and unique recombination and subgenomic and DVG patterns.** Related to Main Figure 5. VTA-infected monolayer RNAs from experiments shown in Fig S3 were analyzed by RNAseq and ViReMA. **(A)** The junction frequency (JFreq) was calculated as the ratio of detected junctions per 1 million mapped nucleotides. **(B)** Junction frequencies were calculated for defective viral genomes (DVGs) and total subgenomic RNAs (sgmRNA) and plotted as the percentage of total mapped junctions. Individual sgmRNA junction frequencies from panel B are shown as the percentage of total mapped junctions for **(C)** sgmRNA 2, **(D)** sgmRNA 3, **(E)** sgmRNA 4, **(F)** sgmRNA 5, **(G)** sgmRNA 6, **(H)** sgmRNA 7, **(I)** sgmRNA 8 , and **(J)** sgmRNA 9. Graphed are individual values, mean, and ± SEM error bars from three independent experiments, n=3. *p<0.05, **p<0.01, ***p<0.001, ****p<0.0001, ns not significant as determined by one-way ANOVA with Dunnett’s multiple comparison test. **(K)** SARS-CoV-2 MA10, **(L)** RAYF, **(M)** AVFS, **(N)** VHVV, and **(O)** YQAV recombination junctions are mapped according to their genomic position (5’ junction site starting position, 3’ junction site sop position) and colored according to their frequency in the population of all mapped junctions. The highest frequencies are purple, and the lowest frequencies are red. Dashed boxes represent clusters of junctions (i) 5’🡪 3’, (ii) mid genome 🡪 3’, (iii) 3’ 🡪 3’, (iv) local deletions, (v) 5’ UTR 🡪 rest of genome. Shown are representative results from one of three independent experiments with similar outcomes.

**Figure S6: Interferon deficiency partially restores SARS-CoV ExoN- replicative fitness in vivo.** Associated with Main Figure 7. **(A)** SARS-CoV MA15 and AAGH virus interferon beta sensitivity. MA-104 cells were pretreated with a dose response of interferon beta 6hr prior to infection with an MOI of 1. Infectious virus production was measured at 48 hpi by plaque assay. Data is from one independent study. **(B)** Schematic of in vivo pathogenesis study in WT C57BL/6 or interferon alpha, beta, gamma receptor knock out mice infected with WT SARS-CoV MA15 (WT N = 27, KO N = 25) or SARS-CoV MA15 AAGH (WT N = 42, KO = 41). **(C)** Body weight loss over time for mice described in “B”. The symbol represents the mean and the error bars represent the standard deviation. **(D)** Viral lung titers for mice described in “B”. The line is at the mean and the error bars represent the standard error of the mean. Asterisks in C and D indicate statistical significance by Two-way ANOVA with Sidak’s multiple comparison test. Data is from three independent studies.

# **Table S1: Sequences of viable saturation mutagenesis variants enriched in passage.** The amino sequence from residue 90-95, the amino acid differences from WT DVEGCH sequence, the percentage in the population and the enrichment score is shown. The enrichment score was generated by comparing the percentage of a given variant at P0 to the percentage of that same variant after 2 passages.

# **Table S2: Coding mutations identified in SARS-CoV-2 AAGH virus stocks.** RNA was extracted from cell monolayers that produced the indicated viral stocks and analyzed by RNAseq. Non-engineered variants are listed that met the following conditions: i) at least 10% frequency, ii) coded for amino acid substitution, iii) not identified in WT stock. Variants that did not meet the 10% frequency cut off were included if they met the conditions listed above for at least one virus stock.

**Table S3: Coding mutations identified in SARS-CoV-2 MA10 nsp14 active site virus stocks.** RNA was extracted from cell monolayers that produced the indicated viral stocks and analyzed by RNAseq. Non-engineered variants are listed that met the following conditions: i) at least 10% frequency, ii) coded for amino acid substitution, iii) not identified in WT stock. Variants that did not meet the 10% frequency cut off were included if they met the conditions listed above for at least one virus stock.
